# A previously unknown feeding mode in millipedes and the convergence of fluid feeding across arthropods

**DOI:** 10.1126/sciadv.abm0577

**Published:** 2022-02-16

**Authors:** Leif Moritz, Elena Borisova, Jörg U. Hammel, Alexander Blanke, Thomas Wesener

**Affiliations:** 1 Zoological Research Museum Alexander Koenig, Leibniz Institute for the Analysis of Biodiversity Change, Section Myriapoda, Adenauerallee 160, 53113 Bonn, Germany.; 2 Institute of Evolutionary Biology and Animal Ecology, University of Bonn, An der Immenburg 1, 53121 Bonn, Germany.; 3 Swiss Light Source, Paul Scherrer Institute, Forschungsstrasse 111, 5232 Villigen PSI, Switzerland.; 4 Institute of Materials Physics, Helmholtz-Zentrum Hereon, Max-Planck-Str. 1, 21502 Geesthacht, Germany.

## Abstract

We report fluid feeding with a sucking pump in the arthropod class Diplopoda, using a combination of synchrotron tomography, histology, electron microscopy, and three-dimensional reconstructions. Within the head of nine species of the enigmatic Colobognatha, we found a pumping chamber, which acts as positive displacement pump and is notably similar to that of insects, showing even fine structural convergences. The sucking pump of these millipedes works together with protractible mouthparts and externally secreted saliva for the acquisition of liquid food. Fluid feeding is one of the great evolutionary innovations of terrestrial arthropods, and our study suggests that it evolved with similar biomechanical solutions convergent across all major arthropod taxa. While fluid-feeding insects are megadiverse today, it remains unclear why other lineages, such as Colobognatha, are comparably species poor.

## Teaser

Fluid feeding also evolved within Diplopoda.

## INTRODUCTION

Arthropods are the most diverse group of animals, and they evolved an immense variety of feeding mechanisms. Fluid feeding is widespread among several arthropod lineages such as tardigrades, onychophorans, arachnids, crustaceans, and insects, and it has been suspected for a small group of millipedes, the Colobognatha (*1*–*3*). Hitherto, the internal morphology of the feeding apparatus and the feeding mechanism of these millipedes have remained largely unknown. To better understand how colobognaths take up food and how fluid feeding evolved in arthropods, a comparison with other suctorial arthropods is essential. Within several arthropod lineages, different pumping mechanisms evolved for the transport of fluids from the exterior into the alimentary canal. All these pumping systems rely on creating negative pressure to draw in liquids. This can be achieved in various ways, like the triradiate sucking pharynx of tardigrades and velvet worms (*4*), by peristaltic contraction of the gut as in Pauropoda (*5*), or by one or several more complex pumping chambers as in arachnids (*6*), parasitic crustaceans (*7*), and many insects (*8*). Complex pumping organs for fluid feeding are most diverse and best studied in fluid-feeding insects, in which they evolved independently in several major lineages contributing to half the insect diversity (*9*, *10*). In most fluid-feeding insects, a proboscis, formed by the mouthparts, is combined with a pumping chamber, which has a similar architecture in several orders (*11*), and might have played a role in the diversification of insects (*12*). Liquids are drawn into the food canal and transported into the foregut by a combination of capillary forces and a pressure gradient created by a volumetric change of the pumping chamber. These pumping chambers are usually formed by modifications of the cibarium, a preoral chamber anterior of the actual mouth, and show a common morphological pattern: The chamber consists of a rigid sclerotized floor, a flexible roof, which is raised by strong dilator muscles to expand the lumen, and anterior and posterior valves or muscles, which direct the flow of fluids. So far, similar structures are unknown from millipedes, which mainly feed on dead plant material with biting-chewing mouthparts (*13*).

**Fig. 1.**
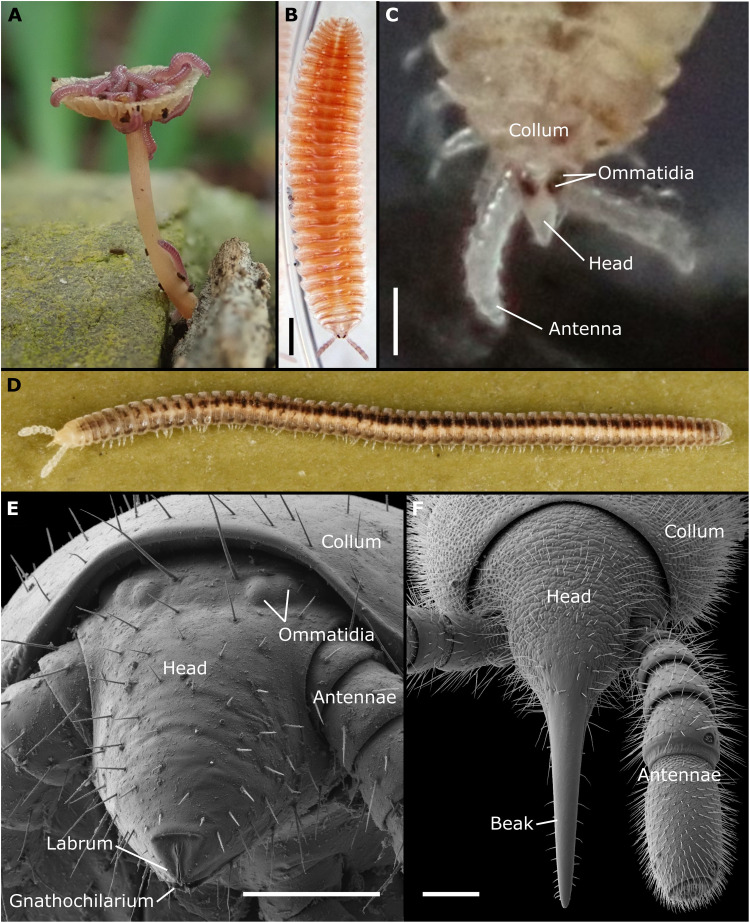
The habitus and external head morphology of suctorial millipedes (Colobognatha). (**A**) *Rhinotus purpureus* (Polyzoniida, Siphonotidae) on a lamellate Basidiomycota in the greenhouse of the Botanical Garden Bonn. (**B**) *Hirudisoma roseum* (Polyzoniida, Hirudisomatidae) from Georgia, habitus. (**C**) *Hirudicryptus canariensis* (Siphonocryptida: Siphonocryptidae) from Tenerife, head. (**D**) *Siphonophora* cf. *zelandica* (Siphonophorida, Siphonophoridae) from New Zealand, habitus; photograph by H. Reip. (**E**) *Siphonetus* sp. (Polyzoniida, Siphonotidae) from New Zealand, fronto-lateral view, scanning electron microscopy (SEM). (**F**) *Siphonethus* sp. (Siphonophorida, Siphonophoridae) from New Zealand, head dorsal view, SEM. Scale bars, 100 μm (B, C, and F) and 20 μm (E).

For the colobognathan millipedes (colobo, reduced; gnathos, jaw), a species-poor remnant group of the Diplopoda, fluid feeding was suspected because of their acuminate heads and the largely reduced or modified mandibles, compared to biting-chewing millipedes (*1*–*3*, *14*, *15*). Nevertheless, the exact mode of fluid uptake is unclear, and a structure similar to the pumping organs of other suctorial arthropods is unknown. Not only their feeding mechanism but also their food source remains enigmatic. Colobognatha often inhabit moist habitats and can be found near fungi (Fig. 1A), and some have been observed to probe rotting plant material (*3*). Therefore, Colobognatha probably feed on algal films, bacterially degraded substances, or fungal hyphae (*16*–*18*). On the basis of their gut content, the latter is the main food source of the Platydesmida (*16*), which might even show external digestion (*18*). All these food sources share a more or less liquid consistency, which requires special adaptations of the feeding apparatus. Compared to fluid-feeding insects and arachnids, the Colobognatha are rather species poor and appear to be a remnant group (*19*) with ~250 species, divided into four groups, Platydesmida, Polyzoniida (Fig. 1B), Siphonocryptida (Fig. 1C), and Siphonophorida (Fig. 1D), which constitute 2% of the extant millipede diversity (*20*). Except for Platydesmida, which show the classic transverse moving mouthpart configuration without a sucking pump and rather feed by “slurping” (*21*, *22*), the head morphology of the other lineages has not been studied. Here, we study the head morphology of representatives of all families within the remaining three colobognathan lineages (table S1). Our results not only show that liquid feeding is also present in millipedes but also compare it to other suctorial arthropods to present a hypothesis on the general feeding mechanism in this group.

## RESULTS

**Fig. 2.**
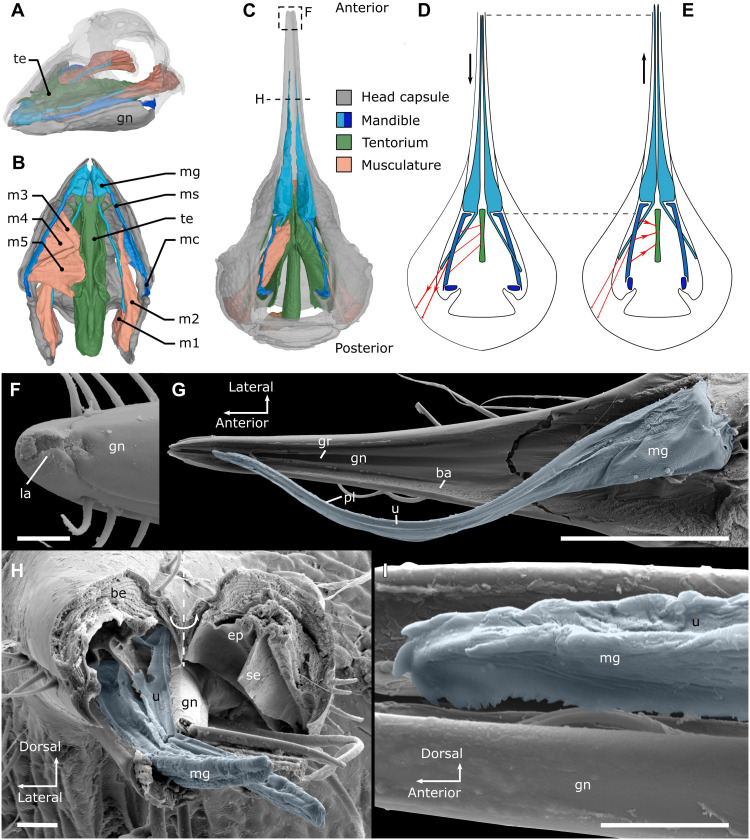
The mandibles of fluid-feeding millipedes. (**A** to **C**) Musculoskeletal system of the mandibles, segmentation based on μCT data, not to scale. (A) *H. roseum* (Polyzoniida), fronto-lateral view. (B) *H. roseum* (Polyzoniida), ventral view with gnathochilarium removed. (C) *Siphonophora* sp. (Siphonophorida), ventral view with gnathochilarium removed, head capsule transparent; the apical tip of the gnathal lobe was not visible in the SR-μCT data. (**D** and **E**) *Siphonophora* sp. schematic representation of mandibular musculature when the mandibles are retracted (D) and protruded (E), arrows indicate movement of gnathal lobe. (**F** to **I**) *Siphonophora* sp., SEM, mandibular gnathal lobe in blue. (F) Tip of beak, ventral view, as indicated in (C). (G) Right mandibular gnathal lobe on top of gnathochilarium, dorsal view, second mandible removed. (H) Cross section through the rostrum, as indicated in (C) showing both mandibular gnathal lobes resting within the gnathochilarium, distal part of rostrum folded to the right. (I) Mandibular gnathal lobe, apical tip, lateral view. Scale bars, 10 μm (F, H, and I) and 100 μm (G). ba, band of cuticular fibers on the gnathochilarium; be, beak; ep, epipharynx; gn, gnathochilarium; gr, groove on inner surface of gnathochilarium; la, labrum; m1 to m5, mandibular muscles; mc, mandibular cardo; mg, mandibular gnathal lobe; ms, mandibular stipes; pl, lamellae on the lateral surface of the gnathal lobe; se, median septum from epipharynx; u, dorsal u-shaped excavation of gnathal lobe.

**Fig. 3.**
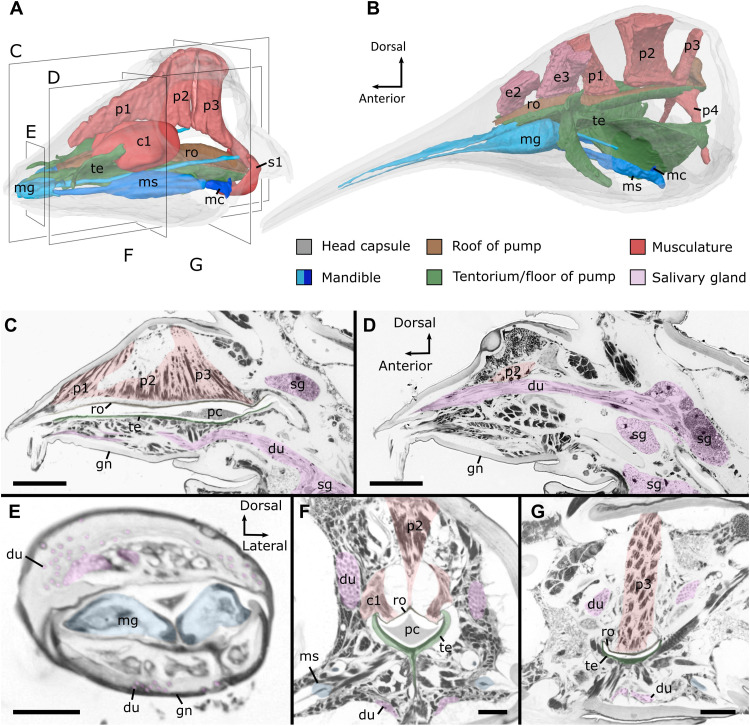
The sucking pump and salivary glands of fluid-feeding millipedes. (**A** and **B**) Sucking pump and its associated musculature, three-dimensional segmentation based on SR-μCT data, fronto-lateral view. (A) *H. roseum* (Polyzoniida), cutting planes of (C) to (G) are indicated. (B) *Siphonophora* sp. (Siphonophorida). (**C** to **G**) *H. canariensis* (Siphonocryptida), histological sections of the head. (C and D) Adult male, sagittal sections: trough pumping chamber (C) and salivary gland (D). (E to G) Adult female, cross sections: trough mandibular gnathal lobe and pores of salivary glands (E), middle portion of pumping chamber (F), and posterior portion of pumping chamber (G). Scale bars, 50 μm (C and D), 20 μm (E), and 10 μm (F and G). c1, compressor muscles; du, ducts of salivary glands; e, epipharyngeal muscles; gn, gnathochilarium; mc, mandibular cardo; mg, mandibular gnathal lobe; ms, mandibular stipes; p, pharyngeal dilator muscles; pc, pumping chamber; ro, roof of pumping chamber; s1, sphincter muscle; sg, salivary glands; te, tentorial complex.

Our histology, scanning electron microscopy (SEM), and micro–computed tomography (μCT) observations showed that the three lineages Polyzoniida, Siphonocryptida, and Siphonophorida share several features regarding their head morphology and their feeding apparatus, which cannot be found in any other non–colobognathan millipedes (*13*, *15*). The heads of the studied specimens are minute and range between 128 and 504 μm in diameter and 162 and 815 μm in length (table S2). They are highly conical, taper anteriorly (Figs. 1, C and E, and 2A), and are even drawn out into a long “beak” in some species (Figs. 1F and 2C and fig. S1). Such a beak is formed by the head capsule and the plate-like lower lip called the gnathochilarium, a defining feature of all millipedes. The gnathochilarium is tightly appressed to the lower margin of the head capsule and the upper lip (labrum), leaving apically only a thin slit (Fig. 1E and fig. S2, A to J) or a circular pore (Fig. 2F), as the functional mouth opening to the preoral chamber. SEM data showed that the labrum and the gnathochilarium carry small pores (fig. S2), which are the external openings of the salivary glands, as evident from histological sections (Fig. 3, C to G). Such pores were absent in *Siphonophora*. The SEM data furthermore showed that the labrum (and the gnathochilarium of the Siphonophorida) carries a median incision, which is lined by teeth (Figs. 1E and 2F and fig. S2).

The paired, largely internalized mandibles consist of three parts: the cardo, the stipes, and the gnathal lobe (Fig. 2, A to C). High-resolution synchrotron (SR)–based μCT data showed that the short cardo, which lacks muscles, articulates with the head capsule and is inclined against an inward projecting protuberance. The long and slender stipes of the studied taxa articulates with the cardo and is equipped with a set of mandibular muscles, which arise mesally from a branched sclerotized endoskeleton, the so-called tentorial complex, and a single muscle arising posteriorly from the head capsule (Fig. 2, B and C, figs. S3 to S11, and table S3). Anteriorly, the stipes articulates with the mandibular gnathal lobe, which is small and triangular in Polyzoniida and Siphonocryptida (figs. S3D and S4D) and extremely elongated and stylet-like in some Siphonophorida (Fig. 2, C and G, and fig. S10). Apically, the gnathal lobes carry larger and smaller teeth, which form a ventral saw-like structure in Siphonophoridae (Fig. 2, H and I, and fig. S12). At its base, the gnathal lobe of all studied taxa gives rise to an apodeme, the gnathal lobe sclerite, to which a single muscle (m1), arising posteriorly from the head capsule, inserts (Fig. 2, B and C, and figs. S3C, S4C, S8B, and S10B).

Behind the mandibular gnathal lobes, the preoral chamber opens into a pumping chamber in all studied taxa (Fig. 3, A and B, and fig. S14). The u-shaped floor of this chamber is thick and high in contrast, which might be due to sclerotization, and it is supported by the tentorial complex, a part of the head endoskeleton in arthropods (Fig. 3). The channel formed by the floor is closed dorsally by a thin roof to which large dilator muscles (p1, p2, and p3), arising from the head capsule, insert (Fig. 3 and fig. S14). In Polyzoniida and Siphonocryptida, additionally, a pair of large compressor muscles (c1) spans above the roof (Fig. 3, A and F, and figs. S3C and S4C). Posteriorly, the pumping chamber opens into the foregut and is surrounded by a well-developed circular muscle (s1), which appears distinct from the general muscular wall of the pharynx in most species (Fig. 3, A and C, and figs. S3C, S4C, and S8B). However, such a sphincter muscle is absent in Siphonophoridae (Fig. 3B and fig. S10).

Fluid feeding in other arthropod groups often relies on passive capillary forces in addition to active suctorial feeding. We assessed the potential for a capillary effect by calculating the equilibrium height for water for Siphonophoridae, which have the longest beak reported here. The lumen of their beak is mesally divided by a septum, resulting in two separated cylindrical food canals (Fig. 2H and fig. S15, A and B). The equilibrium height for water was calculated for two scenarios following Jurin’s law (*23*) to account for morphologies with and without a septum. In the first scenario, the food canal is a single cylinder with a diameter of 15 μm, and in the second scenario, the food canal is subdivided into two cylinders, each with a diameter of 7 μm, reflecting the septum reported above. If the inner surface of the food canal is hydrophilic and the contact angle (θ) is 0° (*24*, *25*) (*10*), the equilibrium height is 1.982 m for the first scenario and 4.248 m for the second scenario. Assuming a less hydrophilic surface and therefore a contact angle of 45°, the equilibrium height is 1.402 and 3.004 m, respectively. Assuming a very weakly hydrophilic surface (θ = 89°), the equilibrium height is 0.035 and 0.074 m, respectively (see Supplementary Text). Given that even theoretical values at the upper and lower extremes of the theoretically possible fluid and surface characteristics result in capillary effects several times higher than the longest beak lengths, it can be assumed that the studied taxa can also access fluids with a higher viscosity such as particles (e.g., from fungi or algae) suspended in saliva or bacterially degraded substances.

## DISCUSSION

### The pumping chamber and feeding mechanism of colobognathan millipedes

**Fig. 4.**
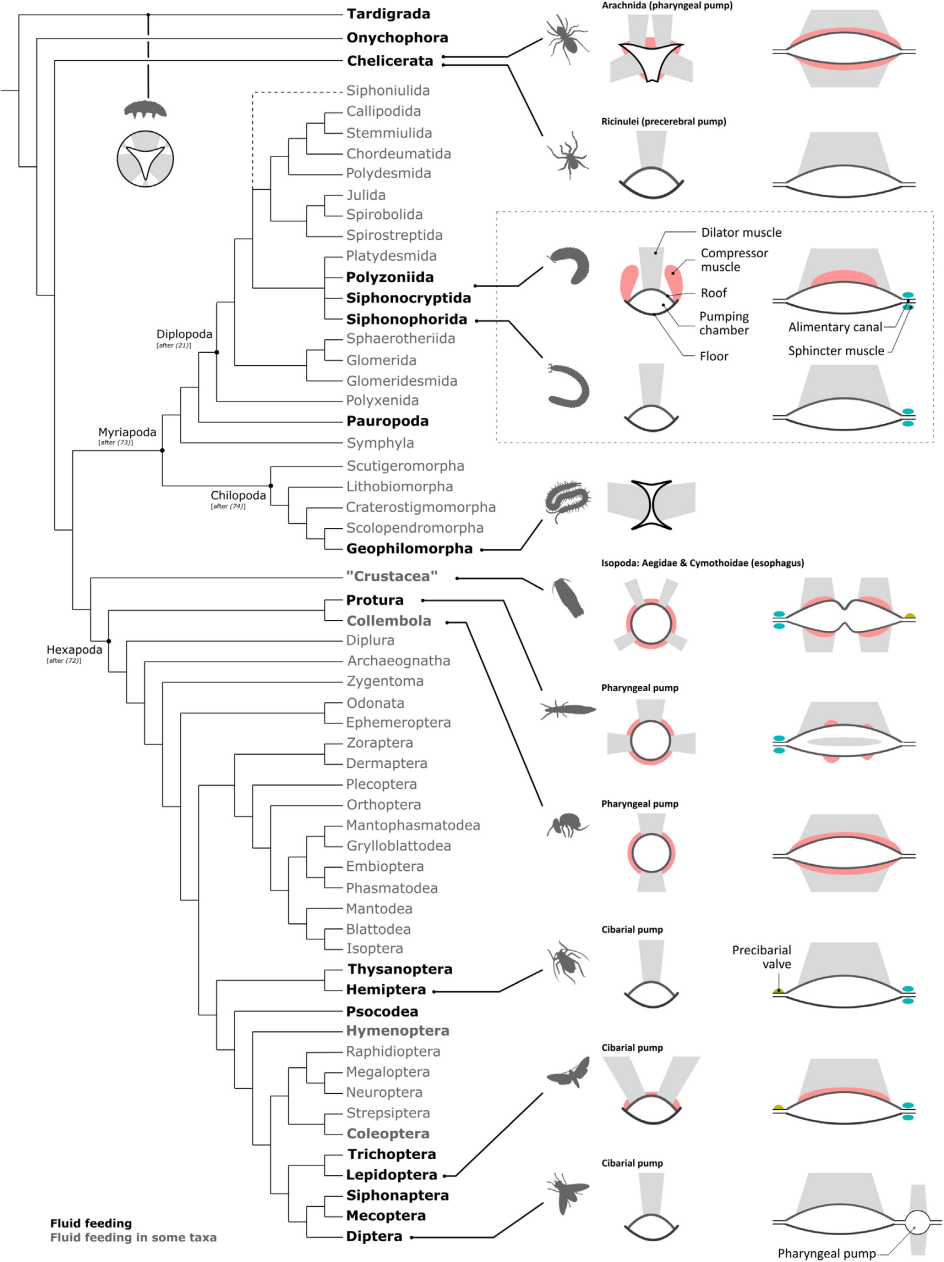
Sucking pumps and their functional components in arthropods. The backbone phylogeny is based on (*71*), internal relationships within Hexapoda according to (*72*), within Myriapoda according to (*73*), within Chilopoda according to (*74*), and within Diplopoda according to (*21*). For references on the sucking pumps in the taxa, see table S4. Schematic and simplified representations of the pumping organs of various arthropods are shown on the right. Cross sections of the pump are shown on the left, and longitudinal sections are shown on the right; for Tardigrada and Geophilomorpha only the cross section is shown. Colors do not indicate homology but functional analogy.

An active pumping mechanism for the intake of liquid food evolved independently in several arthropod lineages (Fig. 4 and table S4), but all share a uniting functional principle: A positive displacement pump forces fluid in or out of a chamber by a change of its volume (*26*, *27*). Tardigrades, onychophorans (*4*), and sea spiders (*28*) have a triradiate sucking pharynx without constrictor muscles, while many arachnids (*6*), springtails (*29*), and proturans (*30*) share a pumping chamber with dorsal and ventral or lateral dilator muscles and surrounding compressor muscles.

Within myriapods, the predatory geophilomorph centipedes might have a pharyngeal pump with a double-anchor cross section and lateral dilator muscles (*31*, *32*), while the minute Pauropoda suck out the contents of fungal hyphae by peristaltic movements of the midgut (*5*). For millipedes (Diplopoda), we demonstrate the presence of a sucking pump and active suction feeding, which resembles that of most fluid-feeding insect lineages even in fine structural details. All studied Colobognatha share a pumping chamber with a thick floor, which is formed by the tentorial complex, and a thin, supposedly flexible roof, to which large dilator muscles adhere. The pumping chamber of millipedes might have been formed by parts of the preoral chamber and the pharynx because epipharyngeal muscles and pharyngeal muscles insert on its roof (see Supplementary Text and table S3).

The basic pattern of the colobognathan pumping chamber resembles that of various suctorial insects like the Hemiptera (*33*, *34*), Lepidoptera (*8*, *35*), some Hymenoptera (*36*, *37*), Diptera (*38*), Siphonaptera (*8*), and Thysanoptera (*11*, *38*, *39*) (Fig. 4 and table S4). These insects all show a cibarium, modified into a pumping chamber with a sclerotized rigid floor and a thin flexible roof, to which large dilator muscles adhere. A similar pumping system with a sclerotized floor and a flexible roof with dilator muscles can also be observed in the precerebral sucking pump of Ricinulei within Chelicerata (*40*). Lepidoptera (*35*, *41*), some Hymenoptera (*36*), and some Coleoptera (*42*) have dorsal compressor muscles spanning above the pumping chamber’s roof, as present in Polyzoniida and Siphonocryptida, while these are absent in other insect lineages and Siphonophorida. This common detailed structure of the pumping chamber in the suctorial millipedes, various suctorial insect lineages, and some Arachnida apparently evolved convergent.

In the studied millipedes, the volume of the sucking pump can increase by the contraction of the large dilator muscles, while the thick ventral wall prevents deformation due to negative pressure buildup. This is also the case in most suctorial insects with a similarly structured pumping chamber (Fig. 4) (*8*, *33*–*38*). During fluid intake, the posterior sphincter muscle closes the sucking pump posteriorly in Polyzoniida, Siphonocryptida, and Siphonorhinidae, similar to Lepidoptera (*35*, *43*). When the sphincter muscle relaxes, the content of the sucking pump is emptied into the foregut passively by the elastic retraction of the dorsal wall in Siphonophorida, as is the case in Hemiptera and Diptera (*34*, *44*, *45*), or actively by the action of muscles dorsally of the chamber, which are only present in Polyzoniida and Siphonocryptida and might function similarly to the compressor muscles spanning across the roof of the pumping chamber in Lepidoptera (*10*, *35*) and in some Hymenoptera (*37*, *46*, *47*) and Coleoptera (*42*, *48*, *49*). A mechanism closing the sucking pump anteriorly to prevent fluid flow out of the mouthparts was reported for butterflies, moths, and Hemiptera (*43*, *44*) but could not be identified in the studied millipedes. Available evidence suggests that, in suctorial millipedes, the filled pump is closed anteriorly by the labrum and gnathochilarium, which can be tightly appressed to each other [fig. S2; (*15*, *50*)]. Fluid intake might be further facilitated by capillary forces acting at the minute slit-like opening of the preoral chamber. The minute opening of the preoral chamber, with an incised labrum, results in capillary forces, which are sufficient to fill even the elongated beak of Siphonophoridae, as is the case in butterflies (*10*). The upper estimate of the height of water that rises within the proboscis of Siphonophorida is more than 4 m for a beak with a diameter of 7 μm, which surpasses the beak length by multiples and suggests that no suction pressure is needed to fill the proboscis. A similar phenomenon was observed in butterflies, where the height of water can range between 14.7 m for a 2-μm diameter and 14.7 cm for a 200-μm food canal (*10*). Considering that the hydrophilic properties of the beak are unknown and that the food might be more viscous, lower values can be expected for Siphonophorida. We suggest that a mixture of capillary forces and active pumping is used to transport liquids into the alimentary canal.

### Protrusion-retraction mechanism of the mandibles

Although Colobognatha have been observed on fungi or probing rotting plant material (*3*), the actual food intake and their internal morphology have not been documented to date. On the basis of the arrangement of muscles and skeletal elements, compared to biting-chewing millipedes and Platydesmida, with mandibles that move in a transverse plane (*13*, *22*, *51*–*53*), the most likely movement of the mandibles in the studied millipedes is a protrusion-retraction through the minute functional mouth opening, similar to the protrusion movement of insect maxillae (*30*, *54*). Therefore, the mandibles can only be used to penetrate surfaces or to loosen particles by scraping or piercing instead of chewing. When the mandible is retracted, the cardo is inclined against a ventral protuberance of the head capsule (Fig. 2D). By contraction of the muscles spanning between mandibular stipes and tentorial complex (m3, m4, m5; Fig. 2), the mandibular base straightens and moves forward, resulting in the protrusion of the gnathal lobe (Fig. 2E). The mandible is retracted by contraction of the muscles spanning from the cranium to the mandibular stipes (m2) and to the gnathal lobe sclerite (m1; Fig. 2D). On the basis of the lengths of the gnathal lobe muscle, the mandibular cardo, and the mandibular stipes, the tips of the gnathal lobes can protrude through the opening of the preoral chamber (see Supplementary Text). In addition, the endoskeleton (tentorial complex) is mesally fused and supports the pharynx and is therefore probably immobile. This contrasts the swinging movement of the tentorium, which is essential for the mandibular abduction in biting-chewing millipedes (*13*, *15*, *51*).

### Externally secreted saliva

In biting-chewing millipedes, the salivary glands open within the preoral chamber each via a single duct (*55*), and the released saliva is involved in the enzymatic digestion of polysaccharides, lipids, and proteins (*56*). In contrast, the salivary glands in fluid-feeding millipedes open via several cuticular tubes and small pores externally nearby the functional mouth opening. These pores and ducts (fig. S13) are a potentially apomorphic character for Colobognatha. For Siphonorhinidae, the release of secretion has been observed from these pores (*50*). Saliva released from the pores might aid in lubrication of the beak, in suspending detached particles for fluid intake, or in external digestion (*18*, *57*). The release of saliva via several small pores spread on the labrum and gnathochilarium, compared to larger amounts via a single opening, might also serve in creating a thin film of saliva instead of larger droplets, which would move away from the conical heads’ tip toward the point of the lowest curvature (*58*, *59*).

### Diversity and evolution of fluid-feeding millipedes

In insects, the evolution of a sucking pump paired with a proboscis might have played a role in their enormous diversification (*12*). While fluid-feeding insects are extremely diverse and represent nearly half of all insect species (*9*, *10*), the fluid-feeding millipedes (Colobognatha) constitute only around 2% of the extant millipede diversity [ca. 250 of the 11,000 described species (*20*)]. In extant samples from tropical forests, less than 6% of millipede abundance is attributed to the Colobognatha [e.g., (*60*)], while they were the dominant millipede group ca. 100 million years ago based on the oldest known remains found in Burmese amber (*61*). The external morphology of these Cretaceous age Colobognatha is almost identical to that of extant representatives (*62*). The lower diversity of extant Colobognatha compared to the megadiverse suctorial insects might be related to the lower dispersal ability and dependence on moist habitats of colobognathan millipedes, which makes them more prone to extinction in changing environments.

Our discovery of a fluid-feeding mode in this group of millipedes shows that similar feeding strategies and biomechanical adaptations toward assessing liquid food evolved across all major arthropod taxa. In this context, the high degree of morphological analogy, even in fine structural details, is remarkable and underlines the strength of selection toward common functional solutions once a new type of food constituted an evolutionary advantage. However, our overview of fluid-feeding strategies across arthropods also suggests that, although suctorial feeding and specialization might have led to diversification in various insect lineages, this is not the case in other arthropod groups. Fluid feeding per se is not a universal driver of diversification.

## MATERIALS AND METHODS

### Taxon sampling and data deposition

The morphology of the head of nine species representing all six higher taxa (families) of the Polyzoniida, Siphonocryptida, and Siphonophorida was studied (table S1). For comparisons to the Platydesmida, already available μCT data (*21*, *22*) were used. All μCT data, segmentations, and digitalized histological data are deposited on Zenodo (https://doi.org/10.5281/zenodo.5215894). Voucher specimens were deposited in the collections of the Zoological Research Museum Alexander Koenig (ZFMK) (table S1). Specimens were examined and dissected with an Olympus Discovery.V12 stereo microscope.

### SR-μCT and three-dimensional segmentation

For SR-μCT, specimens were fixed in Bouin solution (Morphisto, Art.Nr. 12588) or 95% ethanol (EtOH) (table S1) and critical point dried with a Leica EM CPD 300. SR-μCT data were obtained at the Imaging Beamline P05 (IBL) operated by Helmholtz-Zentrum Hereon (*63*–*65*) at PETRA III [Deutsches Elektronen-Synchrotron (DESY), Hamburg, Germany], at the PSI (Paul Scherrer Institut) SLS Beamline TOMCAT-X02DA (Villingen PSI, Switzerland) (*66*), and at the Super Photon ring-8 GeV (SPring-8, Hyogo, Japan) at Beamline BL47XU (table S1) (*67*). Cropping as well as brightness and contrast adjustments of image stacks were done in Fiji ImageJ version 1.50e (*68*). Segmentations were carried out in ITK-SNAP 3.8.0 (*69*) and further processed in MeshLab v2020.07 (*70*) and Blender 2.77 (www.blender.org) for final rendering. Measurements were taken in Blender 2.77 and Fiji ImageJ version 1.50e.

### Histology

Histological sections were obtained for a male and a female *Hirudicryptus canariensis* (Siphonocryptida), which were fixed in Bouin solution and embedded in epoxy resin (Araldite CY212, Agar Scientific Ltd., R1030) following the manufacturer’s protocol. Semi-thin cross and sagittal sections (1 μm) were obtained with a Leica HistoCore NANOCUT R microtome with a DiATOME histo Jumbo diamond blade and stained with 1% toluidine blue (PanReac AppliChem, A3842.0010) for 2 min. The obtained sections were photographed with an Olympus BX61VS light microscope equipped with a VS120-S6-W slide loader system and are deposited at the ZFMK [ZFMK-HIST000002 (*H. canariensis*, female, cross sections) and ZFMK-HIST000003 (*H. canariensis*, male, sagittal sections)].

### Scanning electron microscopy

For SEM, specimens fixed in 70 or 95% EtOH were dehydrated and critical point dried using a Leica EM CPD 300. The specimens were mounted to SEM stubs using conductive tape and sputtered with gold using the Cressington Sputter Coater 108auto. SEM images were obtained with a Zeiss Sigma 300 VP scanning electron microscope at the ZFMK.

### Capillary forces

To estimate the capillarity with Jurin’s law (*23*), the equilibrium height was calculated without and with accounting for the contact angle of the fluid and the food canal (*10*, *24*, *25*), here calculated (see Supplementary Text) for a contact angle of 0° (high hydrophily), 45° (medium hydrophily), and 89° (low hydrophily).

## Supplementary Materials

### This PDF file includes:

Supplementary Text Figs. S1 to S15 Tables S1 to S4 References

## Notes

View/request a protocol for this paper from *Bio-protocol*.
