# Supplementary material for: A previously unknown feeding mode in millipedes and the convergence of fluid feeding across arthropods
Source: Sci Adv. 2022 Feb 16;8(7):eabm0577. doi: 10.1126/sciadv.abm0577 (PMC8849289; doi:10.1126/sciadv.abm0577)
Supplement: Supplementary file 1 — Supplementary Text Figs. S1 to S15 Tables S1 to S4 References [file sciadv.abm0577_sm.pdf]

Science Advances
AAAS

Supplementary Materials for

A previously unknown feeding mode in millipedes and the convergence of fluid feeding across arthropods

Leif Moritz*, Elena Borisova, Jörg U. Hammel, Alexander Blanke, Thomas Wesener

*Corresponding author. Email: moritz.leif@gmail.com

Published 16 February 2022, *Sci. Adv.* **8**, eabm0577 (2022)
DOI: 10.1126/sciadv.abm0577

This PDF file includes:

Supplementary Text
Figs. S1 to S15
Tables S1 to S4
References

Supplementary Text

Head morphology of the Polyzoniida and Siphonocryptida

Head capsule

The head of all studied Polyzoniida (Figs S1B,C, S2A–F) and Siphonocryptida (Fig. S2G) is acuminate, and its posterior part is dorsally concealed by the collum. The head varies in width between 128 and 390 µm and in length between 162 and 510 µm (Table S2). The head is triangular and elongated in *Rhinotus purpureus* (Figs S1C, S2E), triangular and broader in *Hirudisoma roseum* (Figs S1B, S2A) and *Hirudicryptus canariensis* (Fig. S2G) and drop-shaped and broader in *Polyzonium germanicum* (Fig. S2C). The labrum bears a median incision. Several small pores run along the margin of the labrum (Fig. S2A–H), which are the opening of the salivary glands. Pores are also present on the gnathochilarium and are the opening of another pair of salivary glands (Fig. 3C–G).

In Polyzoniida and Siphonocryptida the posterior half of the head capsule is laterally largely occupied by the antennal socket (Figs 1E, S2A–H, S3A, S4A). The epicranium has a dorsal median projection, and paired lateral projections extending into the collum, and serving as muscle attachment site (Figs S3A, S4A). The median projection is rounded in *H. roseum*, *P. germanicum* and *H. canariensis*, while bifurcated in *R. purpureus*. The frons is bulged and clearly set apart from the clypeus in *H. roseum* (Fig. S6A) and *P. germanicum* (Fig. S7A), while in *R. purpureus* the transition of clypeus to frons is even (Fig. S5A). The ommatidia (1+1 in *R. purpureus*; 2+2 in *H. canariensis*; 3+3 in *H. roseum* and *P. germanicum*) and macrosetae (frontal setae) are positioned between the antennal sockets on the frons (Fig. S2A–H). In *H. canariensis* the posterior pair of ommatidia is larger than the anterior, while in *H. roseum* and P. *germanicum* all ommatidia are equal in diameter. In *R. purpureus* the setae are located anterior of the single pair of ommatidia, in *H. canariensis*, *H. roseum* and *P. germanicum*, which have more than one pair of ommatidia the setae are located mesally of the ommatidia (Fig. S2A–H). A median septum is absent. In *Rhinotus purpureus* an internal V-shaped transverse ridge extends between the antennal sockets anterior of the insertion of the macrosetae (Fig. S5A, B). Such a ridge is absent in the other studied taxa. The genae occupies the area lateral and posterior of the antennal socket, serving as articulation to mandibular cardo and gnathochilarium. The ventral margin of the head capsule is coalesced with the gnathochilarium. The genae is only posteriorly well separated from the gnathochilarium forming a small excavation occupied by the mandibular cardo (Figs S3A, S4A, S7D).

Mandible

The mandibles consist of a gnathal lobe, which is well separated from a bipartite base, consisting of cardo and stipes (Figs S3B, D, S4B, D). The long and slender stipes is hidden underneath the genae,150–270 µm in length and 10–25 mm in width, while the cardo and its articulation to the stipes is visible ventrally and laterally, when the head is detached from the body (Figs S3A, S4A). The cardo, which is devoid of muscles, is short and widens posteriorly (hammer-shaped), 30–60 µm in length and 12–20 µm in width (Table S2). The stipes in *R. purpureus* is ca. 7 times as long as the cardo, and constant in width (Fig. S5D). The stipes in *H. roseum* and *P. germanicum* is ca. 5 times as long as the cardo, and the posterior part (where most muscles insert) is widened while the anterior part is rod-like (Figs S4B. S6D, S7D; Table S2). The cardo

is articulated to the head capsule and the gnathochilarium and its dorsal and ventral margin is connected to these via arthrodial membranes (Figs S5E, F, S6E, F, S7F, G). No muscles insert on the mandibular cardo. The triangular gnathal lobe is ca as long as the cardo in all studied taxa and equipped with apical teeth (Fig. S12A, B). The long and slender gnathal lobe apodeme extends nearly to the posterior tip of the stipes in *R. purpureus* (Fig. S3C) and beyond the cardo in *H. roseum* (Fig. S4C) and *P. germanicum*.

Tentorial complex

In all studied species the tentorial complex is fused ventrally of the pharynx, and consist of a transverse bar, an epipharyngeal bar, a hypopharyngeal bar, and a posterior process (Figs S3C, D, S4 C, D). Within the pharynx lays a structure high in contrast in µCT data of *R. purpureus* (Fig. S5A) and *P. germanicum* (Fig. S7A) and low in contrast in *H. roseum* (Fig. S6A). This structure is also visible in the histological sections of *H. canariensis* (Fig. 3C, F) (the nature of this structure remains unknown, it might be an accumulation of food within the pumping chamber). Laterally on both sides, the tentorial complex is connected to the anterior part of the head capsule close to the border of clypeus and labrum via a transverse bar. The short transverse bar passes over into the epipharyngeal bar which extends above the mandibular gnathal lobe. In *H. roseum* the epipharyngeal bar is bifurcated (Figs S3C, S4C, S7C, E). From the point where transverse bar and epipharyngeal bar meet a dorsal process extends, which is long and slender in *R. purpureus* (Fig. S3B) and short and plate-like in *H. roseum* (Fig. S4B) and *P. germancium*. Ventrally the tentorium sets off a short hypopharyngeal bar which runs within the wall of the preoral chamber underneath the mandibular gnathal lobe (Figs S3D, S4D). Posteriorly the posterior processes are fused and extend, as large plate along the whole length of the ventral and lateral pharyngeal wall. Ventrally the posterior process gives rise to a paired ridge, which serves as attachment for the mandibular muscles. The ridges are laterally more or less bowl-shaped and meet posteriorly. In *R. purpureus* (Fig. S3D) the fused ridge separates posteriorly again and sends of a pair of rectangular plates laterally, which are absent in *H. roseum* (Fig. S4D) and *P. germanicum* and *H. canariensis*.

Musculature

For muscles see also Table S3 and Figs S3–S7

A transverse mandibular tendon as muscle attachment site is absent in Polyzoniida and Siphonocryptida. In biting chewing millipedes this collagenous endoskeleton is the main attachment site for mandibular muscles (13, 51–53). In Platydesmida the transverse mandibular tendon is reduced to a small bridge spanning between the tentorial posterior processes and does not serve as attachment site for mandibular muscles (22).

Antennal muscles:

Median septum antennal muscle (a1): Mesally from posterior margin of head capsule (O) to posterior margin of first antennomere (I).

Median antennal muscle (a2): Tentorial posterior process posterior of a3 (O) to posterior margin of first antennomere (I).

Lateral antennal muscle (a3): Tentorial posterior process anterior of a2 (O) to anterior margin of first antennomere, lateral of a4 (I).

Anterior antennal muscle (a4): Tentorial posterior process anterior of a2 (O) to anterior margin of first antennomere median of a3 (I).

Gnathochilarial muscles:
Anterior median gnathochilarial muscle (g1): Ventral ridge of tentorial complex close to hb, anterior of g3, mesal of p2 (O) to gnathochilarium anterior of g3, mesal of g2 (I)
Anterior lateral gnathochilarial muscle (g2): Ventral ridge of tentorial complex close to hb, lateral of p1 (O) to gnathochilarium lateral of p1 (I).
Median gnathochilarial muscle (g3): Ventral ridge of tentorial complex posterior of g1 (O) to gnathochilarium posterior of g1, mesal of g2.
Median gnathochilarial muscle (g4): Ventrally on anterior mesal tip of tentorial complex between g1 (O) to posterior margin of gnathochilarium between g5 (I). Unpaired.
Mesal median gnathochilarial muscle (g5): Tentorial posterior process ventral ridge between g1 and g6, In R. purpureus from rectangular lateral projections of the posterior process (O) to gnathochilarium between g1 and g6, lateral of g4, mesal of 75 (I).
Posterior lateral gnathochilarial muscle (g7): Gnathochilarium posterior margin lateral of g6 and g3 (O) to gnathochilarium lateral of g3 (I).
Posterior median gnathochilarial muscle (g8): Posterior ventral surface of tentorial posterior process, posterior of g5 (O) to gnathochilarium posterior margin lateral of g4 (I)

Mandibular muscles:
Gnathal lobe sclerite muscle (m1): Lateral posterior projection of head capsule dorsally of m2 (O) to gnathal lobe apodeme (I).
Dorsal mandibular stipes muscle (m2): Lateral posterior projection of head capsule ventrally of m2 (O) to median margin of mandibular stipes ventrally of m5 (I).
Anterior mandibular stipes muscle (m3): Tentorial posterior process ventrally of m4 (O) mandibular stipes anterior of m4 (I).
Median mandibular stipes muscle (m4): Tentorial posterior process anterior of m5 (O) to stipes between M3 and m5 occupying most of the posterior half of stipes (I).
Posterior mandibular stipes muscle (m5): Tentorial posterior process posterior of m4 (O) to mandibular stipes posterior of m4 (I).

Pharyngeal muscles:
Anterior pharyngeal dilator muscle (p1): Head capsule mesally, anterior of p2 (O) to dorsal pharyngeal wall, anterior of p2 (I).
Median pharyngeal dilator muscle (p2): Head capsule mesally, between p1 and p3 (O) to dorsal pharyngeal wall, between p1 and p3 (I). In PR from Head capsule internal transverse ridge between antennal bases (O).
Posterior pharyngeal dilator muscle (p3): Head capsule mesally, posterior of p2 (O) to dorsal pharyngeal wall, posterior of p2 (I).
Sphincter muscle (s1): From collagenous tendons from median process of head capsule (O) around ventral pharynx posterior of tentorium (I)
Transverse pharyngeal muscle (c1): Ventral spanning over ventral wall of sucking pump, crossing p1.

Tentorial muscles:

Anterior tentorial muscle (t1): Head capsule anterior of t2 and t3 (O) to dorsal margin of small dorsal plate of tentorial complex anterior of t4 close to tentorial transverse bar (I).
Median tentorial muscle (t2): Head capsule posterior of t1 and median of t4 (O) to dorsal margin of tentorial complex antero-mesal of t1 (I). In RP from extension of tentorial complex.
Dorsal tentorial muscle (t5): Head capsule lateral of t2 and posterior of t1 (O) to posterior tip dorsal projection from epipharyngeal bar of tentorial complex, posterior of t1 (I).

Salivary gland
The pores on the labrum of the Polyzoniida and Siphonocryptida (Fig. S2A–H) are the opening of the salivary glands. From the pores on the labrum tubes (conductive canals) run as bundles to a paired salivary gland, which lays within the anterior body-rings dorsally of the intestine and anterior of the brain (Fig. 3D–G; S13A). The pores on the gnathochilarium give rise to tubes, which run in bundles to a paired salivary gland, which lay ventrally of the intestine (Fig. 3C). We suggest that the gland opening on the labrum of Polyzoniida and Siphonocryptida is homologous to the anterior salivary glands of other millipedes, in which it is located anterior of the brain and opens on the epipharynx (55). Like the anterior salivary gland in other millipedes the dorsal salivary gland of the Polyzoniida and Siphonocryptida is located anterior of the brain, which is located in the anterior body-rings as shown for Polyzoniida (75). The ventral salivary gland, which opens on the gnathochilarium of Siphonocryptida, might be homologous to the posterior salivary gland of other millipedes, which is located in the anterior body-rings and opens on the hypopharynx in these (55).

Head morphology of the Siphonophorida

Head capsule
The overall shape of the head capsule of *Siphonorhinus* sp. and *Siphonophora* sp. is similar except for the anterior portion, which is triangular in *Siphonorhinus* (Figs S1E, S2I) and extends into a long beak in *Siphonophora* spp. (Figs 1F, S1E, S2K). The length of the head varies between 560 and 815 µm and its width between 270 and 504 µm. The head is broad at its base and nearly cylindrical. Its posterior opening is cylindrical with the gula fused to the head capsule. The insertion of the antennae opens anteriorly. Anterior of the antennae base the head narrows (Fig. S1D, E). The labrum of *Siphonorhinus* sp. is triangular and incised anteriorly (Fig. S2J) as in the Polyzoniida, while in *Siphonophora* sp. the head is drawn out into a long and slender beak (Figs 1F, S2K), which is also incised at its tip (Fig. S2L). An incision can also be found on the gnathochilarium (Fig. S2J, L). The incisions are lined by teeth (Fig. S12G, H). An incisura lateralis is absent. A short, paired projection arises from the ventral posterior margin of the head and runs into the head capsule, where it articulates against the mandibular cardo (Figs S8C, S9D, S10C, S11D). The genae is broad and bends mesally underneath the head forming a rim on which the gnathochilarium lays (Figs S9C, S11C). A median septum is absent.

Mandible
The mandibles consist of a gnathal lobe, which is well separated from a bipartite base, consisting of cardo and stipes (Figs S8C, S9D, S10C, S11D). The stipes is long (130–216 µm in length) and slender (16-26 µm in width) and ca. 10 times longer than wide in *Siphonophora* sp. and 8 times in *Siphonorhinus* sp.. The cardo, which is devoid of muscles is short (ca. 1/6 of the length of the

stipes in *Siphonophora* sp. and 1/3 in *Siphonorhinus* sp.), and posteriorly widening, nearly triangular (Table S2) with 26–66 µm in length and 14–31 µm in width. It is articulated to the projection of the head capsule (Figs 2C, S9D, S11D). In *Siphonorhinus* sp. the gnathal lobe is triangular in shape elongated, and with 276 µm ca as long as the stipes (Fig. S8C). In *Siphonophora* sp. the gnathal lobe is stylet-like and 344 µm long, with a broad base and a pointed tip; it is ca two times as long as the stipes and extends along the whole length of the beak (Figs 2G, S12E, F). The gnathal lobe apodeme is ca as long as the stipes and rod-like (Figs S8A, S10A). In *Siphonophora* spp. the elongated gnathal lobes extends into the beak (Figs 2C–E, S12E, F) formed by the elongated head capsule and the gnathochilarium, which form a closed tube with an inner diameter of ca.15 µm and an apical ventral opening (Figs 2H, S11E, S15I, K, S16A, B). In specimens with a lowered gnathochilarium the tips of the gnathal lobes are visible externally and rest within the gnathochilarium (Fig. S12C, D). The gnathal lobes are separated by a median septum, which arises dorsally from the epipharynx and extends along the whole length of the beak (Figs 2H, S12E), resulting in a median separation of the beak into two compartments, each with a diameter of ca 7 µm. The gnathochilarium carries a longitudinal groove running on its internal surface (endochilarium) underneath the gnathal lobes opposite of the epipharyngeal median septum (Figs 2G, S12F, H). Apically the gnathal lobes are laterally flattened and carry terminal teeth as well as a row of small teeth on their ventral margin (Figs 2I, S12J). Dorsally the gnathal lobes have a u-shaped excavation, which runs along their entire length (Figs 2G, H, S12K). Laterally, the gnathal lobes are covered by flat lamellae, which furcate distally (Fig. S12L) and face a distinct band of elongated cuticular fibers running along the inner lateral surface of the gnathochilarium (Fig. S12I).

Tentorial complex

The tentorial complex of the Siphonophorida consists of rigid transverse bars, thin and rod-like epipharyngeal bars, hypopharyngeal bars and large plate-like posterior processes (Figs S8B, C, S10A, C). The transverse bar arches above the mandibular gnathal lobe and gives rise to a stout ridge, which projects anteriorly (Fig. S8B) and might correspond to the frontal process. From the point where transverse bar and epipharyngeal bar meet, a projection extends dorsally into the head capsule; this projection is rod-like in *Siphonorhinus* sp. (Fig. S8A), while shorter and stout in *Siphonophora* sp. (Fig. S10A). Ventrally of the preoral chamber the epipharyngeal bars are fused mesally, as well as the anterior portion of the posterior processes (Figs S8C, S9B, S10C, S11B). More posteriorly the posterior processes diverge and form distinct, well separated plates. Between the posterior processes arises the sclerotized ventral wall of the tube-like pumping chamber (Figs S8C, S10C).

Musculature

For muscles see also Table S3 and Figs. S8-S11.

A transverse mandibular tendon is absent in Siphonophorida, as discussed above for Polyzoniida and Siphonocryptida.

Antennal muscles:

Median septum antennal muscle (a1): Laterally from posterior margin of head capsule (O) to posterior margin of first antennomere (I).

Median antennal muscle (a2): Tentorial posterior process ventrally of a3 (O) to posterior margin of first antennomere (I).
Lateral antennal muscle (a3): Tentorial posterior process dorsally of a2 (O) to anterior margin of first antennomere, lateral of a4 (I).
Anterior antennal muscle (a4): Dorsal margin of tentorial posterior process between a3 and a5 (O) to anterior margin of first antennomere, dorsally of a4 (I).
Posterior antennal muscle (a5): Dorsal margin of tentorial posterior process, anterior of a4 (O) to posterior ventral corner of first antennomere (I).

Epipharyngeal muscles:
Anterior lateral epipharyngeal m. (e1): Mesally on cranium, anterior of e2, lateral of e3 (O) to epipharyngeal sclerite (I). Only in Siphonorhinus sp.
Median epipharyngeal m. (e2): Mesally on cranium, between e2 and e3 (O) to epipharynx between e2 and e3 of e2 (I).
Posterior epipharyngeal m. (e3): Mesally on cranium, posterior of e2 (O) to epipharynx posterior of e2 (I).

Gnathochilarial muscles:
Anterior median gnathochilarial muscle (g1): Hypopharynx, mesal of g2 (O) to gnathochilarial mentum mesal of g2 (I).
Anterior lateral gnathochilarial muscle (g2): hypopharynx lateral of g1 (O) to gnathochilarial stipes anterior of g7 (I).
Gnathochilarial muscle (g3): Anterior part of posterior process, ventral margin (O) to mentum, posterior of g1 (I).
Median gnathochilarial muscle (g4): Mesally on Hypopharynx (O) to gnathochilarial mentum, Mesal of g8 (I). unpaired.
Gnathochilarial muscle (g5): Tentorial posterior process ventral margin, posterior of g8 (O) to gnathochilarial stipes (I).
Posterior lateral gnathochilarial muscle (g7): lateral on gula anterior margin (O) to gnathochilarial stipes lateral of g3 (I). Absent in Siphonophora sp.
Posterior median gnathochilarial muscle (g8): Tentorial posterior process, ventral margin anterior posterior of g4 (O) to gnathochilarial mentum, posterior portion (I).

Mandibular muscles:
Gnathal lobe sclerite muscle (m1): Posterior part of cronium dorsally of m2 (O) to gnathal lobe apodeme (I).
Dorsal mandibular stipes muscle (m2): Postoccipitla flange ventrally of m2 (O) to median dorsal margin of mandibular stipes ventrally of m5 (I).
Anterior mandibular stipes muscle (m3): Anterior tentorial posterior process between m4a and m4b (O) mandibular stipes anterior of m4 (I). Fibers running anteriorly. Only ma in Siphonophora sp.
Dorsal & ventral Median mandibular stipes muscle (m4a/b): Tentorial posterior process anterior of m5, dorsal (m4a) and ventrally (m4b) of m3 (O) to stipes between M3 and m5 occupying most of the posterior half of stipes (I). Fibers running posteriorly.

Anterior mandibular cardo muscle (m5): Tentorial posterior process posterior of m4 (O) to anterior portion mandibular cardo posterior of m4 in *Siphonorhinus*, to posterior portion of stipes in *Siphonophora* (I).

Pharyngeal muscles:
Anterior pharyngeal dilator muscle (p1): Headcapsule mesally, anterior of p2 (O), passes anterior of brain to dorsal pharyngeal wall, anterior of p2 (I).
Median pharyngeal dilator muscle (p2): Headcapsule mesally, between p1 and p3 (O), passes through brain to dorsal pharyngeal wall, between p1 and p3 (I).
Posterior pharyngeal dilator muscle (p3): Headcapsule mesally, posterior of p2 (O), passes posterior of brain to dorsal pharyngeal wall, posterior of p2 (I).
Lateral pharyngeal dilator muscle (p5): Tentorial posterior process (O) to lateral pharyngeal wall (I); only present in *Siphonorhinus*.
Ventral postoccipital flange pharyngeal dilator (p7): Ventral corner of postoccipital flange (O) to ventral pharyngeal wall (I).
Sphincter muscle (s1): From collagenous tendons from median process of head capsule (O) around ventral pharynx posterior of tentorium (I)

Tentorial muscles:
Anterior tentorial muscle (t1): Head capsule anterior of t3 (O) to frontal process of tentorial complex (I).
Dorsal tentorial muscle (t2): Head capsule lateral posterior of t1 (O) to dorsal projection from epipharyngeal bar of tentorial complex (I)
Posterior tentorial muscle (t3): Laterally from neck phragma (O) to posterior tip of tentorial posterior process (I).

Salivary gland

The pores, which are arranged in a field on the labrum of Siphonorhinidae, are the opening of a paired salivary gland, which lays within the head anterior of the brain (Fig. S13 B). We suggest that this salivary gland is homologous to the dorsal salivary gland in Polyzoniida and Siphonocryptida and to the anterior salivary gland of other millipedes (Hifnawi & Seifert 1973). In Siphonophoridae no such pores have been observed.

Morphology of the sucking pump

In all studied specimens of the Polyzoniida, Siphonocryptida, and Siphonophorida a pumping chamber with a volume of 0.003679–0.9256 x10^{-3} µl (Table S2) is located within the head behind the mandibular gnathal lobes (Figs 3A, B, S14). The sucking pump consists of (a) a thick floor, (b) a thin roof, equipped with (c) dilator muscles, (d) a posterior sphincter muscle, and (e) in the Polyzoniida and Siphonocryptida a dorsal set of muscles, which might act as compressor muscles (Figs 3, S15). The latter could not be found in the Siphonophorida (Figs 3B, S15G-L). (a) The thick floor of the sucking pump is u-shaped and supported by the branched tentorial complex (the endoskeleton of the head), which is fused in the midline (Fig. 3). Several muscles, which run to the mandibles, head capsule (cranium), antennae and lower lip (gnathochilarium), are associated with the tentorial complex. (b) The roof of the sucking pump is formed by a

thinner supposedly flexible cuticle. (c) Several strong dilator muscles, which originate from the clypeus and frons of the cranium, insert on the roof of the pumping chamber. In the Siphonophorida additional dilator muscles originating from the tentorial posterior process and from the gula insert ventrolateral on the posterior region of the pumping chamber. (d) Posteriorly the pumping chamber narrows and the transition of the sucking pump into the foregut is surrounded by a circular sphincter muscle in Polyzoniida, Siphonocryptida and the Siphonophorida family Siphonorhinidae (Figs 3, S14). In the other Siphonophorida family Siphonophoridae (*Siphonophora* spp.) this sphincter muscle cannot be identified, but the pharynx is curved in an S-shape. (e) In the Siphonocryptida and Polyzoniida a paired muscle inserts on the ventral wall of the sucking pump lateral of the dilator muscles, which is absent in representatives of the Siphonophorida (Figs 3A, S3C, S4C).

Protrusion of the mandible in *Siphonophora*

For the cockroach *Periplaneta germanica* it has been shown that the fiber length of the mandibular abductor muscle changes by 23–35% during biting (76). Assuming a similar change in length in the mandibular muscle m1 and that this muscle is fully contracted in the examined specimen (ZFMK-MYR-F8-Spring8) with a length of 110 µm and 108 µm, this gives a maximum increase in length of 25–38.5 µm.

Assuming that the mandibular cardo and stipes form a straight line when the mandible is fully extruded this would result in a distance of 151 µm and 156 µm between the apical tip of the stipes and the proximal tip of the cardo when straightened (Fig. 2E), compared to 122 µm and 130 µm in the retracted position (Fig. 2D). This means that the upper limit the gnathal lobe can be pushed forward by, is 29 µm, which would be sufficient to protrude the gnathal lobe tip beyond the tip of the beak.

Capillarity

Following Jurin's law (23) and assuming the parameters of water, the equilibrium height of fluid within the food canal is calculated for two scenarios: In the first scenario (#1) the food canal is a single cylinder with a diameter of 15 µm (r ≈ 0.0000075 m); In second scenario (#2) the food canal is subdivided into two cylinders with a diameter of 7 µm (r ≈ 0.0000035). As parameters the surface tension (σ = 0.0728 N/m) and density of water (ρ 998.23 kg/m) and the gravitational acceleration (g =9.81 m/s2) were used.

A) Here the equilibrium height was calculated assuming a contact angle of θ = 0° with the following formula (24, 25):

$H = 2\sigma/\rho g r$

(#1) for d ≈15 µm (r ≈ 0.0000075 m):

H = 2*0.0728/998.23*9.81*0.0000075

H = 0.1456/0.073444772

H=1.982 m

(#2) For d ≈ 7 µm (r ≈ 0.0000035):

H = 2*0.0728/998.23*9.81*0.0000035

H = 0.1456/0.034274227

H=4.248 m

B) Here the equilibrium height for the contact angles $\theta = 45°$ and $\theta = 89°$ was calculated with the following formula (10):

$$H = 2\sigma\cos\theta/(\rho g r)$$

(#1) For $\theta = 45°$:

H = 2*0.0728*cos(45)/998.23*9.81*0.0000075

H = 0.102954747/0.073444772

H=1.402 m

For $\theta = 89°$:

H = 2*0.0728*cos(89)/998.23*9.81*0.0000075

H = 0.002541070/0.073444772

H= 0.035 m

(#2) For $\theta = 45°$:

H = 2*0.0728*cos(45)/998.23*9.81*0.0000035

H = 0.102954747/0.034274227

H=3.004 m

For $\theta = 89°$:

H = 2*0.0728*cos(89)/998.23*9.81*0.0000035

H = 0.002541070/0.034274227

H=0.074 m

The phylogeny of the Colobognatha

The phylogenetic relationship of the four colobognathan taxa is portrayed as unresolved polytomy in Fig. 4, as has been inferred by Blanke & Wesener (21) based on morphological data. The only other phylogenetic hypotheses for the Colobognatha, which includes Siphonocryptida (all other phylogenies (e.g. (77)) are lacking this crucial group) has been proposed by Sierwald & Bond (78). Their total evidence analyses resolves Siphonocryptida as sister group to Platydesmida, but lacks molecular data for Siphonocryptida. Thus, the grouping is supported by a single morphological character, the mid-dorsal suture (their character 38). Siphonocryptid millipedes were classically treated as taxa within Polyzoniida (e.g. (79)), but Shelley (80) elevated the Siphonocryptida to order level, based on an unpublished phylogenetic analysis, which retrieved the Siphonocryptida as sister group to Platydesmida and not as closest relative of Polyzoniida. This classification has been widely accepted in the myriapodological community (e.g. (20, 21, 78)). Therefore, any phylogeny of the colobognathan taxa, which does not include Siphonocryptida, is treated as unresolved or at least questionable. The paraphyly of Polyzoniida + Siphonocryptida needs to be reexamined, as both taxa share several unique characters.

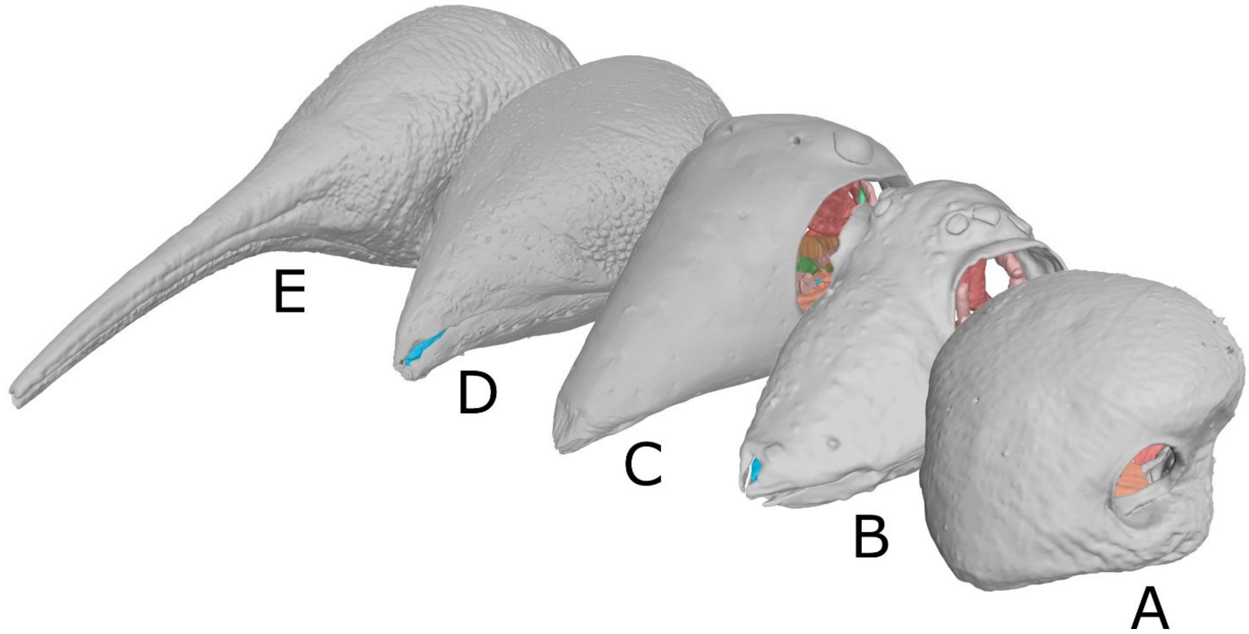

Fig. S1.

The head shape of the Colobognatha, fronto-lateral view, segmentation based on SR-μCT data, not to scale. **(A)** *Dolistenus* sp. (Platydesmida: Andrognathidae). **(B)** *Hirudisoma roseum* (ZFMK-MYR11195) (Polyzoniida: Hirudisomatidae). **(C)** *Rhinotus purpureus* (ZFMK-MYR11197) (Polyzoniida: Siphonotidae). **(D)** *Siphonorhinus* sp. (F9) (Siphonophorida: Siphonorhinidae). **(E)** *Siphonophora* sp. (F8) (Siphonophorida: Siphonophoridae).

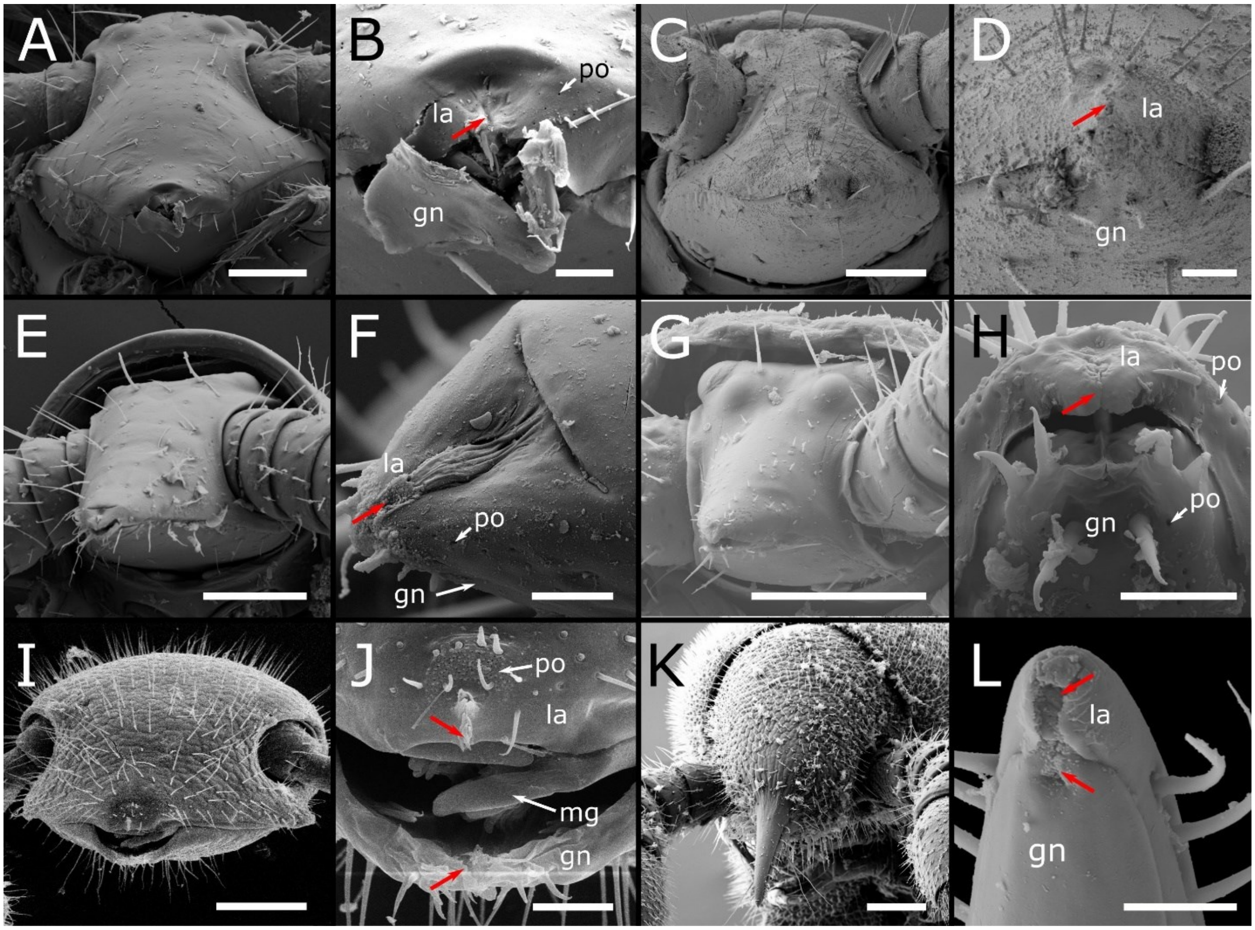

Fig. S2.

Head (A, C, E, G, I, K) and labrum (B, D, F, H, J, L) of the Polyzoniida Siphonocryptida and Siphonophorida. Red arrows indicate the incision of labrum and gnathochilarium. **(A, B)** *Hirudisoma roseum* (ZFMK-MYR7255). **(C, D)** *Polyzonium germanicum* (ZFMK-MYR9888). **(E, F)** *Rhinotus purpureus* (ZFMK-MYR10052). **(G, H)** *Hirudicryptus canariensis* ZFMK-MYR8906). **(I, J)** *Siphonorhinus* sp. (MNHG). **(K, L)** *Siphonophora* cf. *zelandica* (ZFMK-MYR11173). **Scale**: A, C, E, G, I, K= 100 µm, B, D, J = 20 µm, F, H, L = 10 µm. **Abbreviations:** gn = gnathochilarium, la = labrum, mg = mandible gnathal lobe, po = pores.

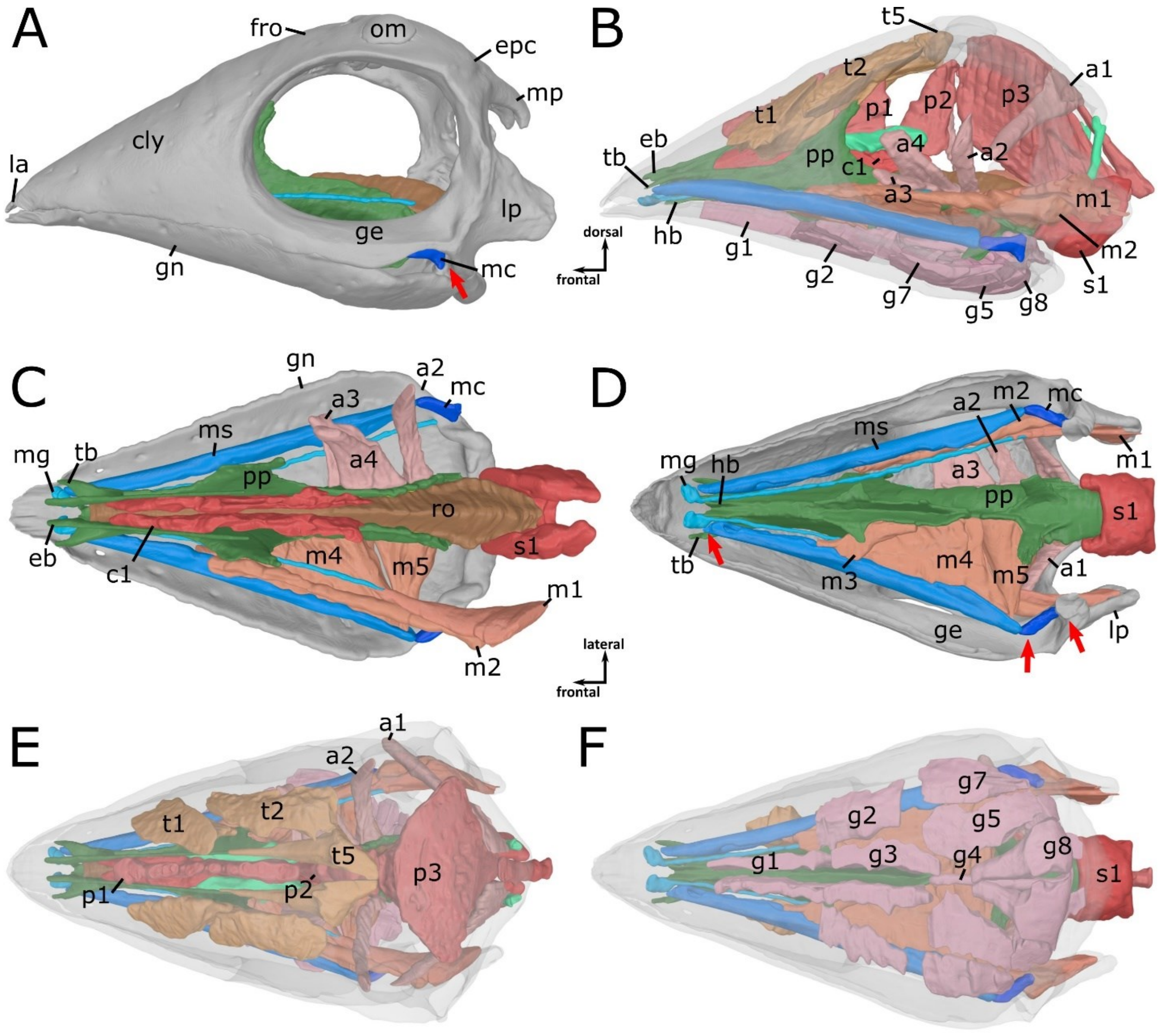

Fig. S3.

Head morphology of *Rhinotus purpureus* (Pocock, 1894) (ZFMK-MYR11197), 3D segmentation. Arrows indicate articulation of the mandible. **(A)** External morphology, lateral view. **(B)** Internal morphology and musculature, lateral view. **(C)** Mandibular and antennal musculature, dorsal view, head capsule removed. **(D)** Mandibular and antennal musculature, ventral view, gnathochilarium removed. **(E)** Tentorial and pharyngeal musculature, dorsal view. **(F)** Gnathochilarial musculature, ventral view. Not to scale. **Abbreviations:** a# = antennal musculature; c1 = compressor muscle, cly = clypeus, eb = tentorial epipharyngeal bar, epc = epicranium, fro = frons, g# = gnathochilarial musculature, ge = gena, gn = gnathochilarium, hb = tentorial hypopharyngeal bar, la = labrum, lp = lateral projection of the epicranium, m# = mandibular musculature, mc = mandibular cardo, mg = mandibular gnathal lobe, mp = median projection of the epicranium, ms = mandibular stipes, p# = pharyngeal musculature, ph = pharynx, pp = tentorial posterior process, ro = roof, s1 = sphincter muscle, t# = tentorial

musculature, tb = tentorial transverse bar. The gnathal lobes could not be fully segmented based on the SR-µCT data.

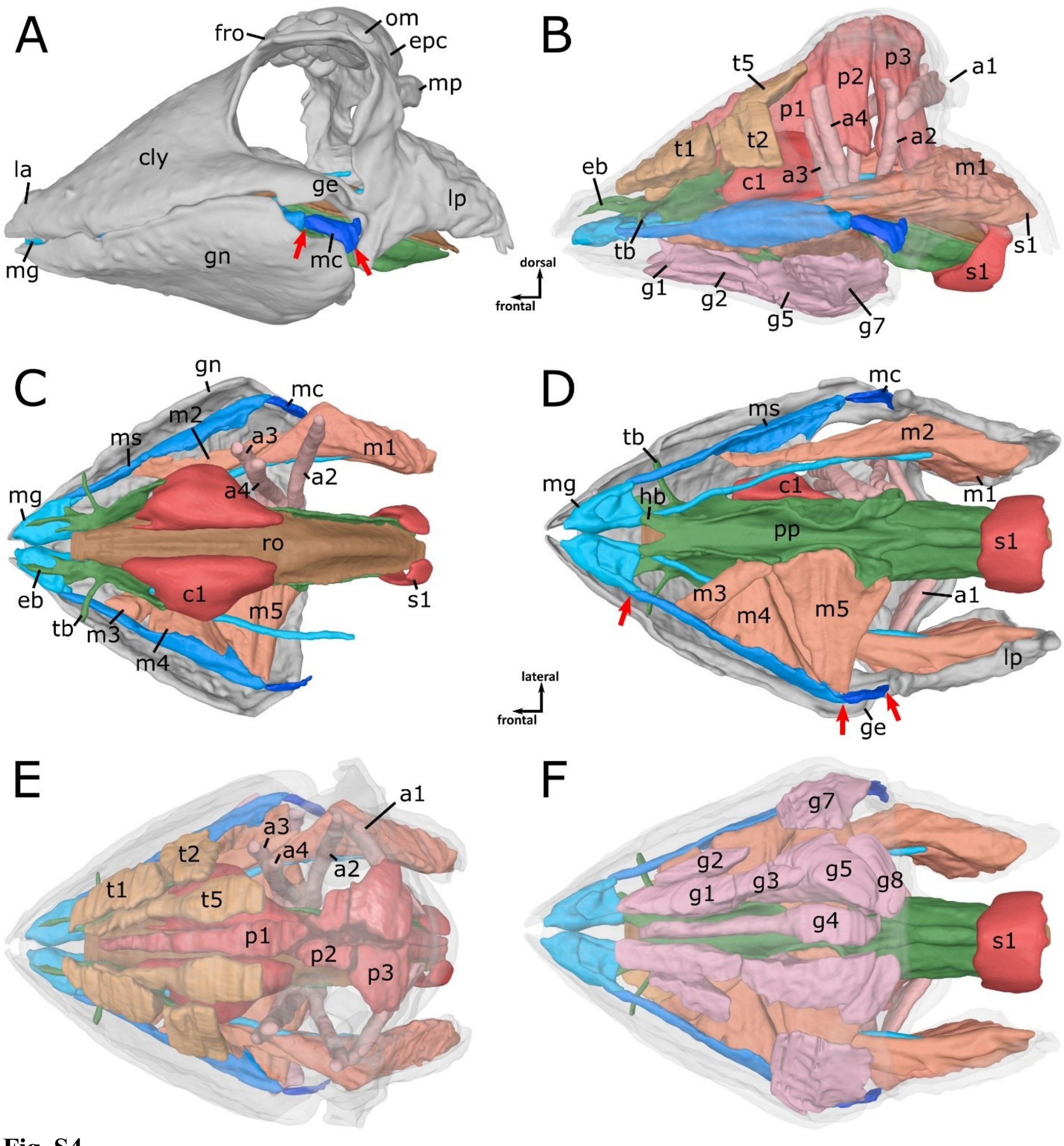

Fig. S4.
Head morphology of *Hirudisoma roseum* (Victor, 1839) (ZFMK-MYR11195), 3D segmentation. Arrows indicate articulation of the mandible. **(A)** External morphology, lateral view. **(B)** Internal morphology and musculature, lateral view. **(C)** Mandibular and antennal musculature, dorsal view, head capsule removed. **(D)** Mandibular and antennal musculature, ventral view, gnathochilarium removed. **(E)** Tentorial and pharyngeal musculature, dorsal view. **(F)** Gnathochilarial musculature, ventral view. Not to scale. **Abbreviations:** a# = antennal musculature; c1 = compressor muscle, cly = clypeus, eb = tentorial epipharyngeal bar, epc =

epicranium, fro = frons, g# = gnathochilarial musculature, ge = gena, gn = gnathochilarium, hb = tentorial hypopharyngeal bar, la = labrum, lp = lateral projection of the epicranium, m# = mandibular musculature, mc = mandibular cardo, mg = mandibular gnathal lobe, mp = median projection of the epicranium, ms = mandibular stipes, p# = pharyngeal musculature, ph = pharynx, pp = tentorial posterior process, ro = roof, s1 = sphincter muscle, t# = tentorial musculature, tb = tentorial transverse bar.

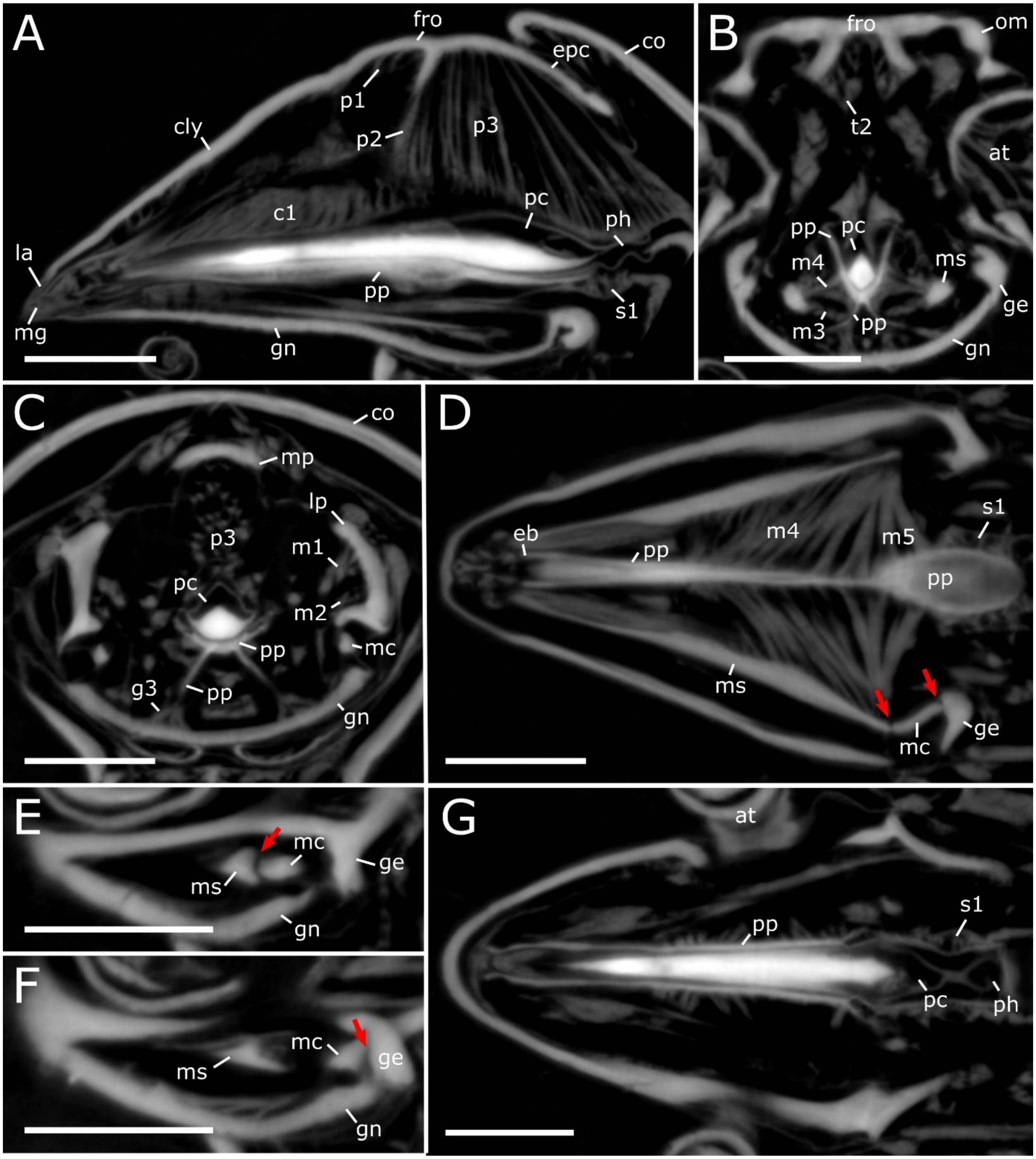

Fig. S5.
Head morphology of *Rhinotus purpureus* (Pocock, 1894) (ZFMK-MYR11197), sections based on SR-µCT data. Arrows indicate articulation of the mandible. **(A)** Sagittal-section through midline of head. **(B)** Cross-section through head at level of antennae. **(C)** Cross-section through posterior part of head. **(D)** Transversal-section through mandibular base. **(E, F)** Sagittal-section through articulation of mandibular stipes and cardo. **(G)** Transversal-section through tentorial complex. **Scale** = 100 µm. **Abbreviations:** at = antenna, cly = clypeus, co = collum, eb = tentorial epipharyngeal bar, epc = epicranium, fro = frons, ge = gena, gn = gnathochilarium, la = labrum, m# = mandibular musculature, mc = mandibular cardo, mg = mandibular gnathal lobe,

ms = mandibular stipes, , p# = pharyngeal musculature, pc = pumping chamber, ph = pharynx, pp = tentorial posterior process, s1 = sphincter muscle, tb = tentorial transverse bar.

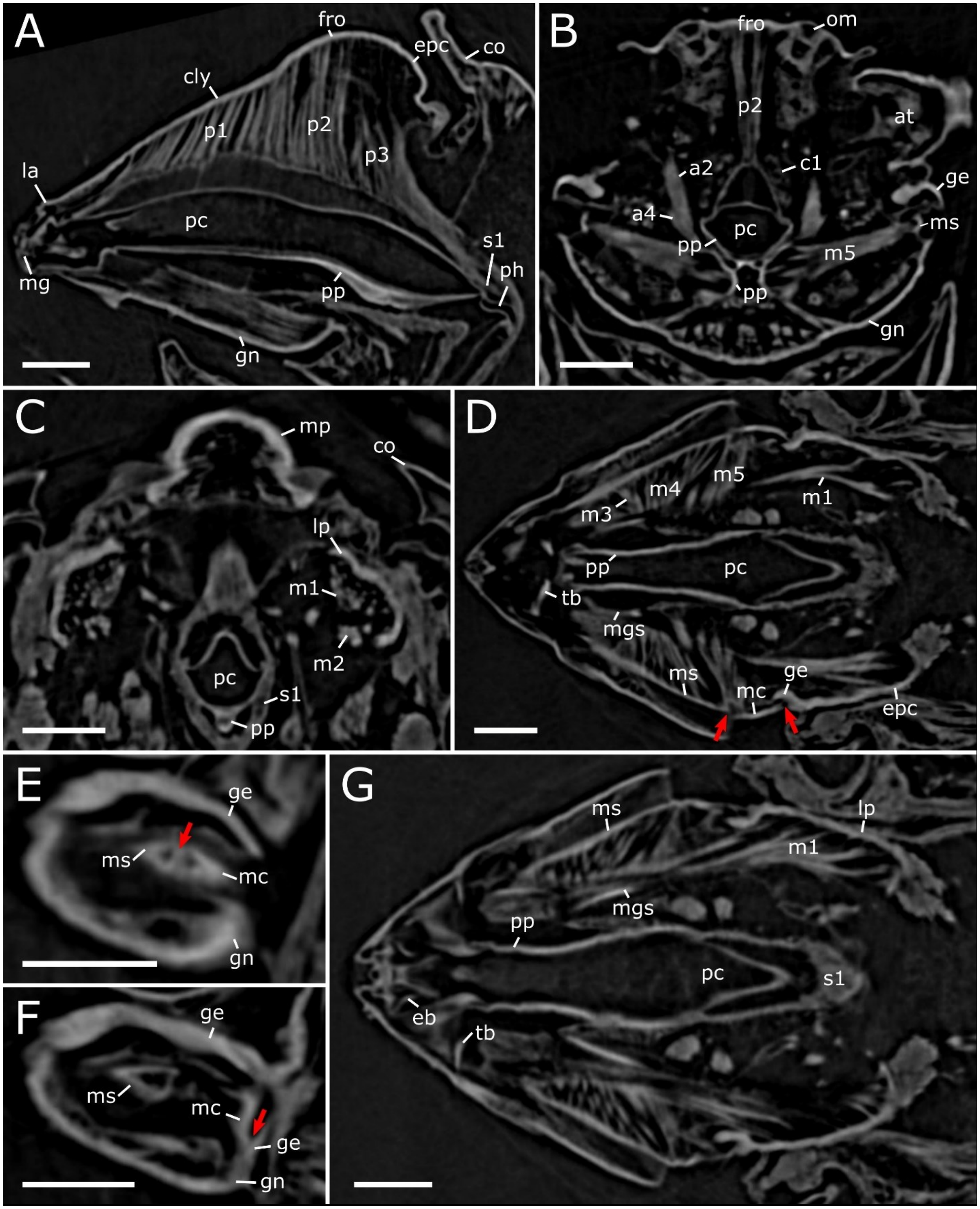

Fig. S6.

Head morphology of *Hirudisoma roseum* (Victor, 1839) (ZFMK-MYR11195), sections based on SR-µCT data. Arrows indicate articulation of the mandible. **(A)** Sagittal-section through midline of head. **(B)** Cross-section through head at level of antennae. **(C)** Cross-section through posterior part of head. **(D)** Transversal-section through mandibular base. **(E, F)** Sagittal-section through

articulation of mandibular stipes and cardo. **(G)** Transversal-section through tentorial complex. **Scale** = 100 µm. **Abbreviations:** at = antenna, cly = clypeus, co = collum, eb = tentorial epipharyngeal bar, epc = epicranium, fro = frons, ge = gena, gn = gnathochilarium, la = labrum, m# = mandibular musculature, mc = mandibular cardo, mg = mandibular gnathal lobe, mgs = mandibular gnathal lobe sclerite, ms = mandibular stipes, , p# = pharyngeal musculature, pc = pumping chamber, ph = pharynx, pp = tentorial posterior process, s1 = sphincter muscle, tb = tentorial transverse bar.

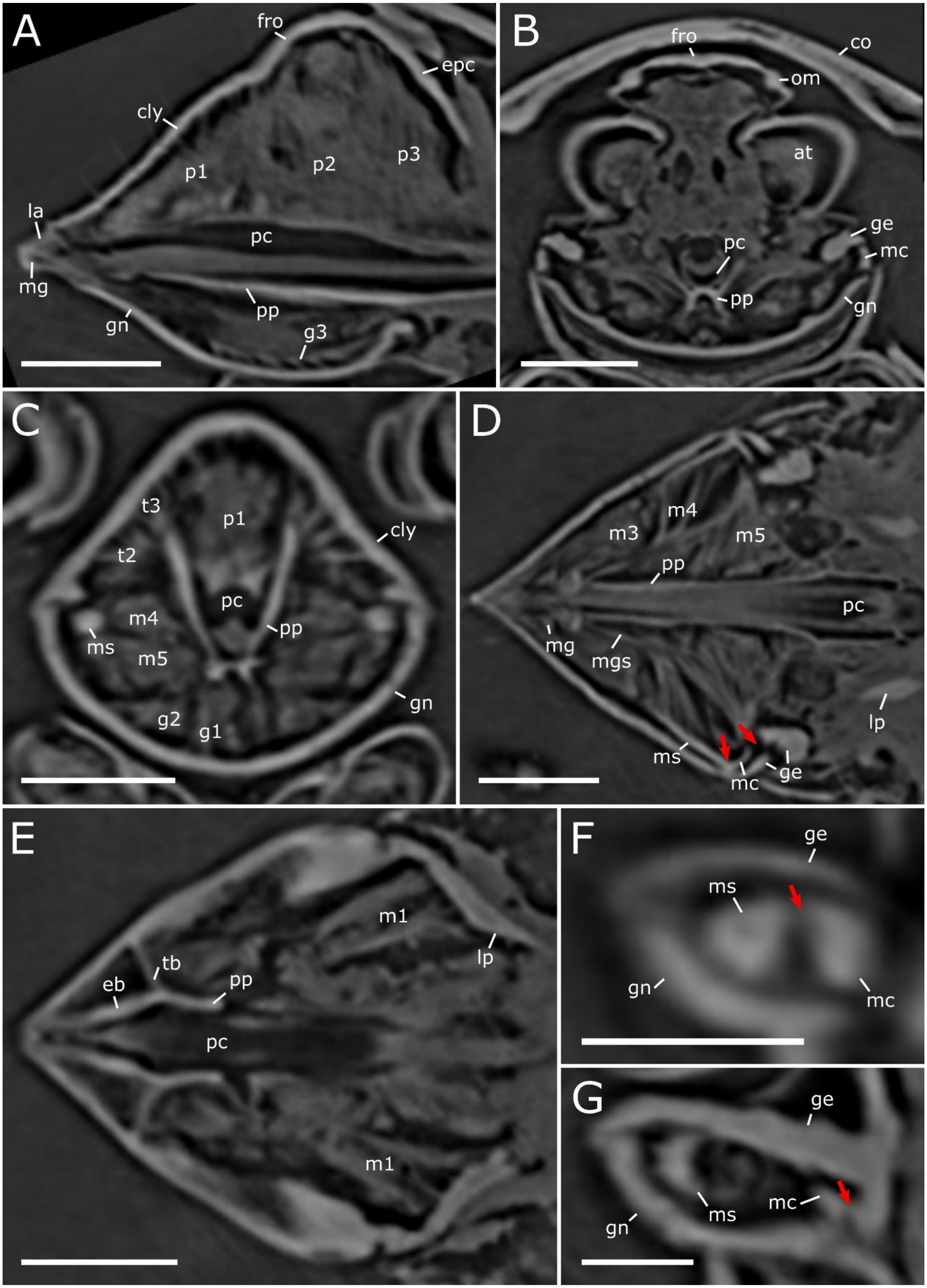
A
fro
epc
cly
p1
p2
p3
la
pc
mg
pp
gn
g3
B
fro
co
om
at
ge
pc
mc
pp
gn
C
t3
p1
cly
t2
pc
m4
pp
ms
m5
gn
g2
g1
D
m4
m3
m5
pp
pc
mg
mgs
lp
ms
mc
ge
E
m1
tb
lp
eb
pp
pc
m1
F
ge
ms
gn
mc
G
ge
mc
gn
ms

← **Fig. S7.**

Head morphology of *Polyzonium germanicum* Brandt (ZFMK-MYR11196), sections based on SR-µCT data. Arrows indicate articulation of the mandible. **(A)** Sagittal-section through midline of head. **(B)** Cross-section through head at level of antennae. **(C)** Cross-section anterior of antennae. **(D)** Transversal-section through mandibular base. **(E)** Transversal-section through tentorial complex. **(F, G)** Sagittal-section through articulation of mandibular stipes and cardo. **Scale**: A – E = 100 µm, F, G = 50 µm. **Abbreviations:** at = antenna, cly = clypeus, co = collum, eb = tentorial epipharyngeal bar, epc = epicranium, fro = frons, ge = gena, gn = gnathochilarium, la = labrum, m# = mandibular musculature, mc = mandibular cardo, mg = mandibular gnathal lobe, mgs = mandibular gnathal lobe sclerite, ms = mandibular stipes, , p# = pharyngeal musculature, pc = pumping chamber, ph = pharynx, pp = tentorial posterior process, tb = tentorial transverse bar.

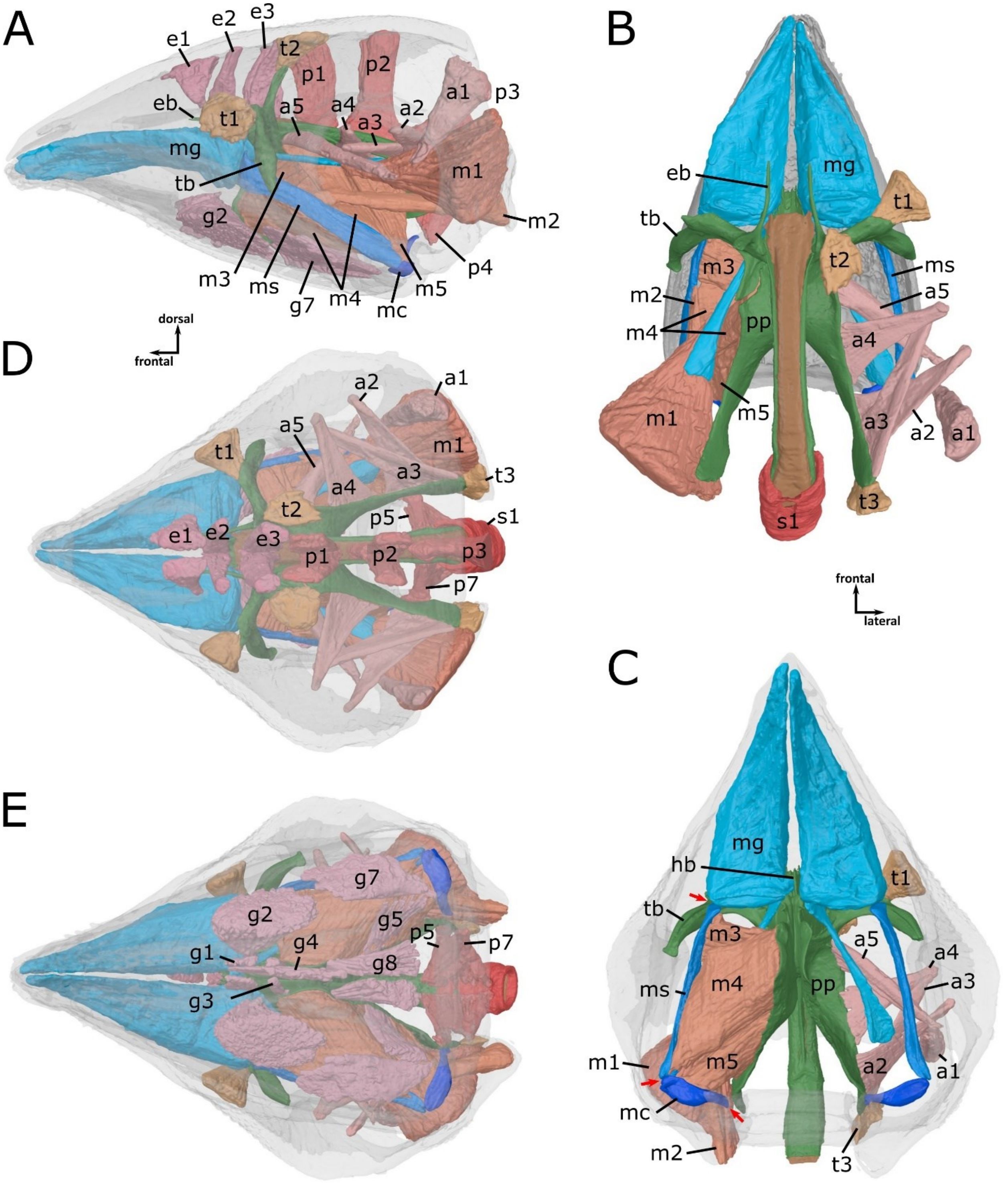

Fig. S8.
Head morphology of *Siphonorhinidae* sp. (F9), 3D segmentation. Arrows indicate articulation of the mandible. **(A)** Lateral view. **(B)** Mandibular and antennal musculature, dorsal view, head capsule removed. **(C)** Mandibular and antennal musculature, ventral view, gnathochilarium removed. **(D)** Dorsal view. **(E)** Ventral view. Not to scale. **Abbreviations:** a# = antennal musculature; eb = tentorial epipharyngeal bar, g# = gnathochilarial musculature, gn = gnathochilarium, hb = tentorial hypopharyngeal bar, lp = lateral projection of the epicranium, m#

= mandibular musculature, mc = mandibular cardo, mg = mandibular gnathal lobe, mp = median projection of the epicranium, ms = mandibular stipes, p# = pharyngeal musculature, ph = pharynx, pp = tentorial posterior process, s1 = sphincter muscle, st = stylet, t# = tentorial musculature, tb = tentorial transverse bar.

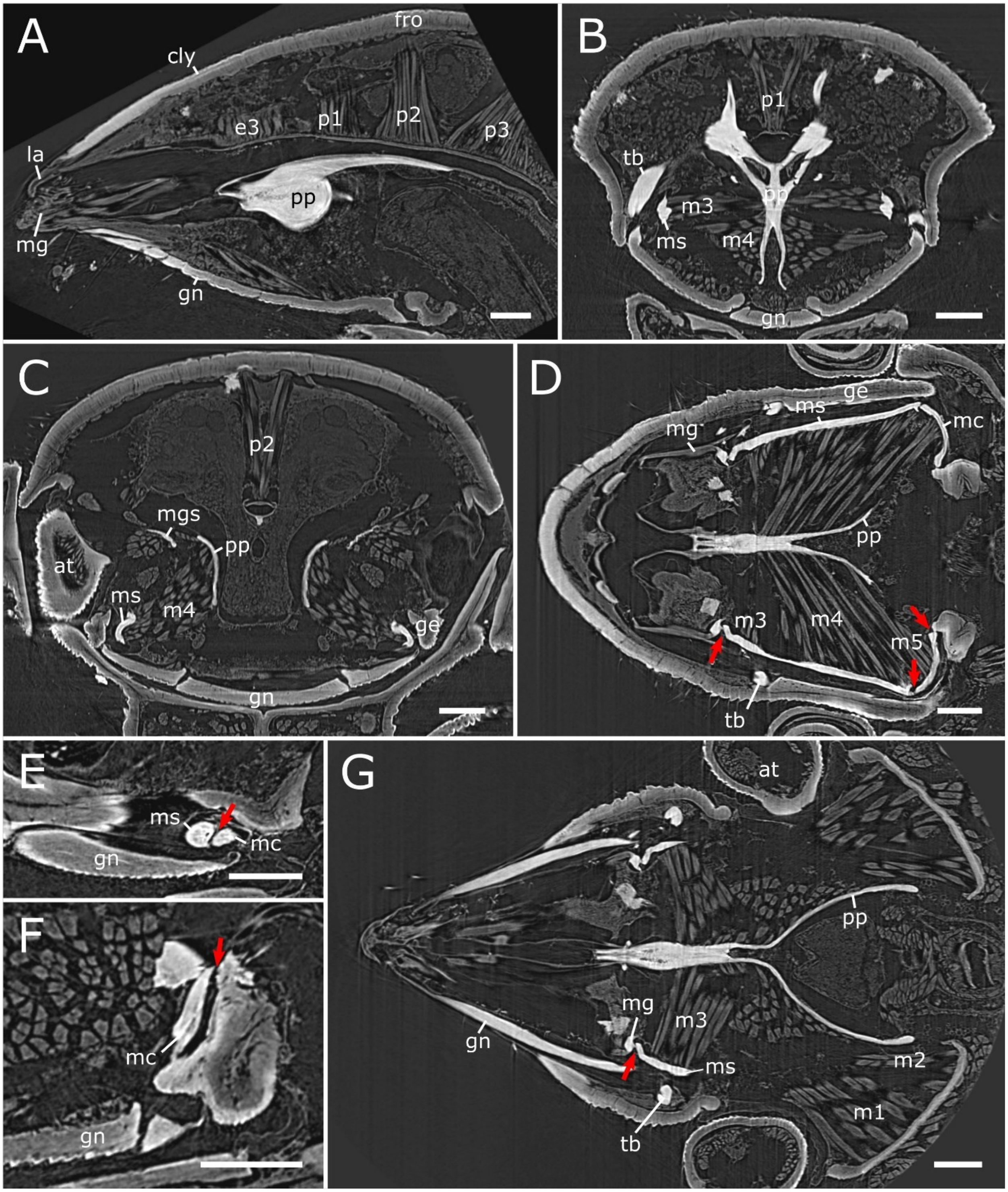

Fig. S9.
Head morphology of *Siphonorhinus* sp.(F9), sections. Arrows indicate articulation of the mandible. **(A)** Sagittal-section through midline of head. **(B)** Cross-section anterior of antennae. **(C)** Cross-section through head at level of antennae. **(D)** Transversal-section through mandibular base. **(E)** Transversal-section through tentorial complex. **(F, G)** Sagittal-section through articulation of mandibular stipes and cardo. **Scale** = 50 µm. **Abbreviations:** at = antenna, cly =

clypeus, co = collum, eb = tentorial epipharyngeal bar, epc = epicranium, fro = frons, ge = gena, gn = gnathochilarium, la = labrum, m# = mandibular musculature, mc = mandibular cardo, mg = mandibular gnathal lobe, mgs = mandibular gnathal lobe sclerite, ms = mandibular stipes, , p# = pharyngeal musculature, pc = pumping chamber, ph = pharynx, pp = tentorial posterior process, s1 = sphincter muscle, st = stylet, tb = tentorial transverse bar.

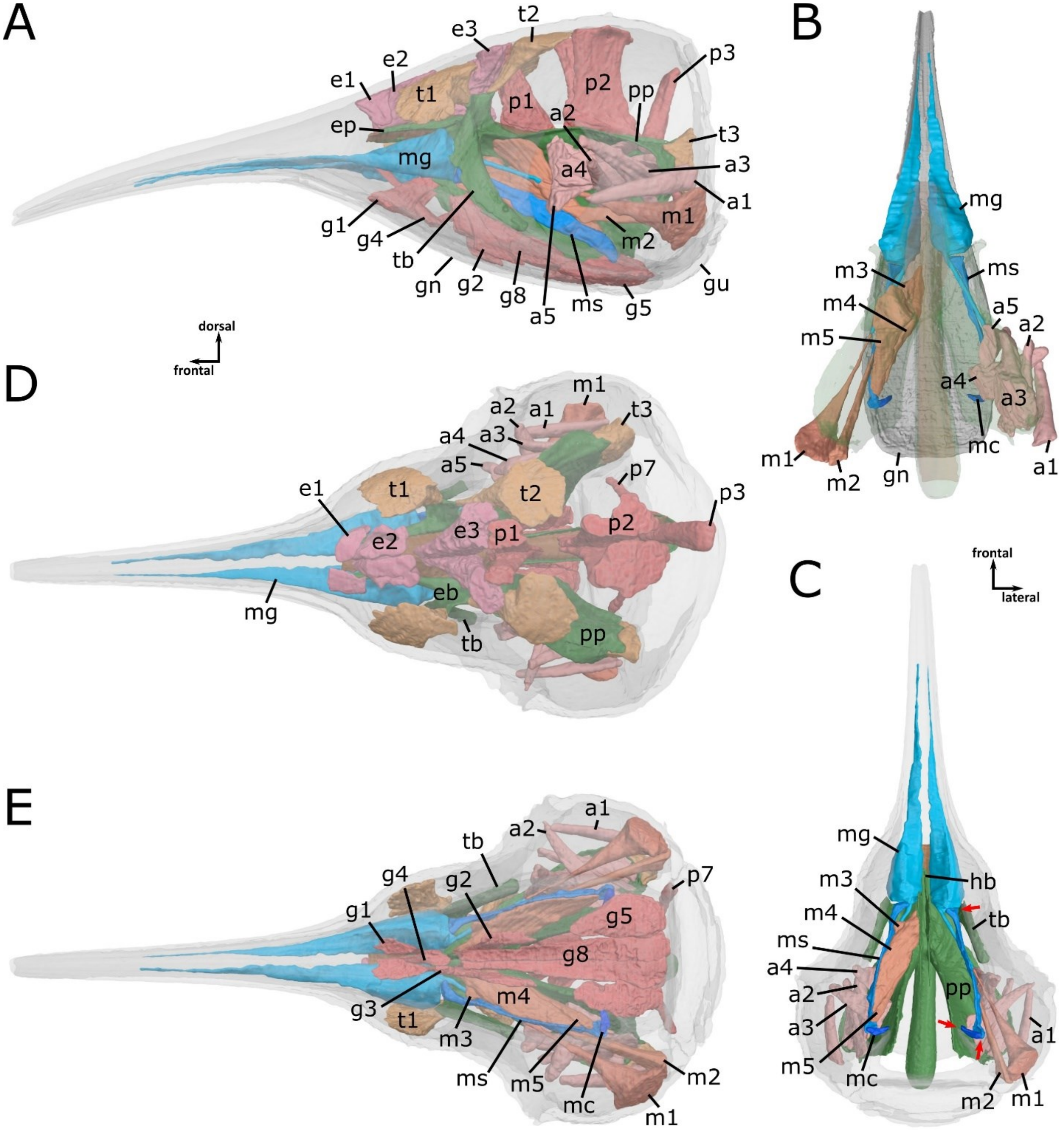

Fig. S10.
Head morphology of *Siphonophora* sp. (F8), 3D segmentation. Arrows indicate articulation of the mandible. **(A)** Lateral view. **(B)** Tentorial, mandibular and antennal musculature, dorsal view, head capsule removed. **(C)** Mandibular and antennal musculature, ventral view, gnathochilarium removed. **(D)** Dorsal view. **(E)** Ventral view. Not to scale. **Abbreviations:** a# = antennal musculature; eb = tentorial epipharyngeal bar, g# = gnathochilarial musculature, ge = gena, gn = gnathochilarium, hb = tentorial hypopharyngeal bar, lp = lateral projection of the epicranium, m# = mandibular musculature, mc = mandibular cardo, mg = mandibular gnathal lobe, mp = median projection of the epicranium, ms = mandibular stipes, p# = pharyngeal

musculature, ph = pharynx, pp = tentorial posterior process, st = stylet, t# = tentorial musculature, tb = tentorial transverse bar.

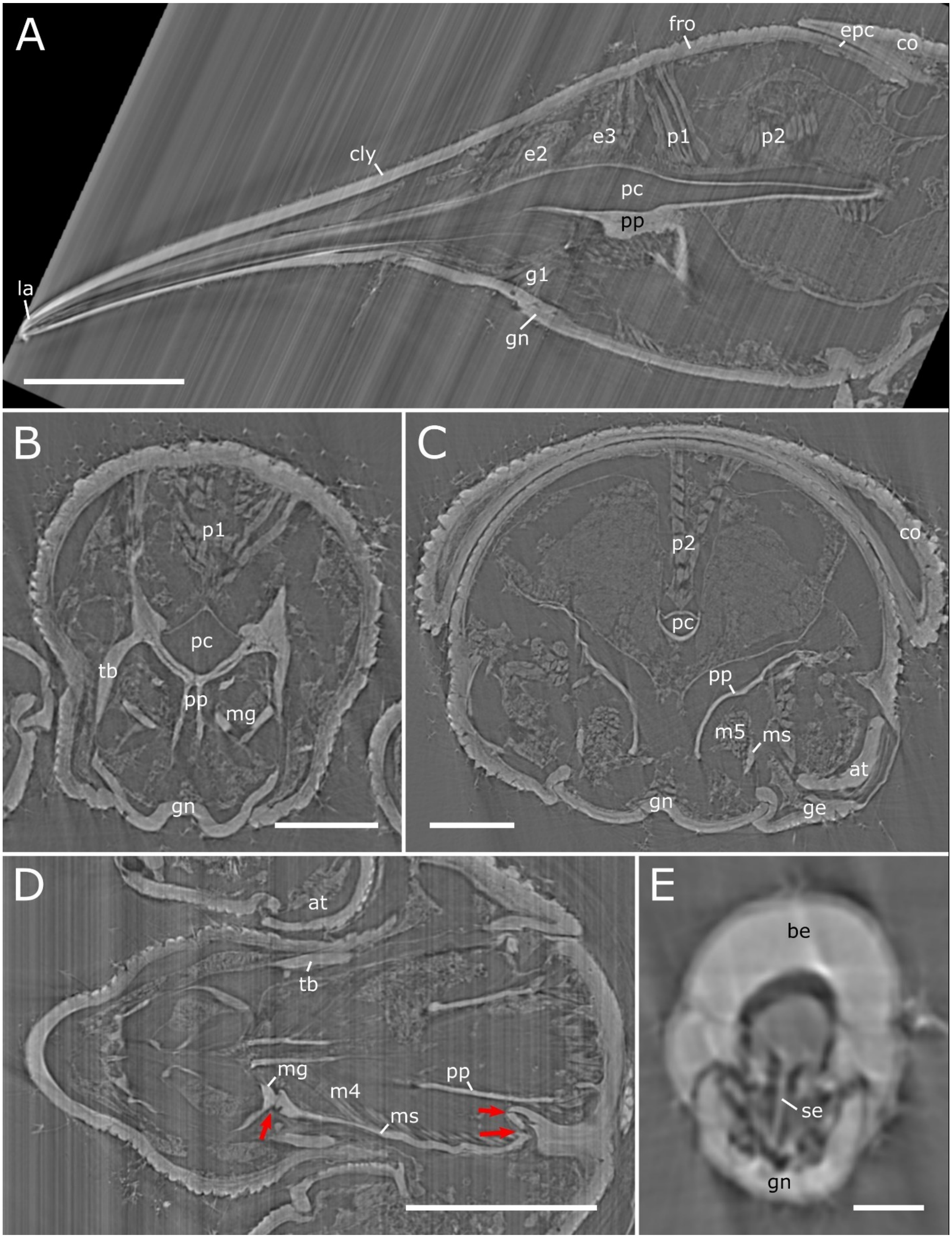
A
fro
epc
co
e3
p1
p2
cly
e2
pc
pp
la
g1
gn
B
p1
pc
tb
pp
mg
gn
C
co
p2
pc
pp
m5
ms
at
gn
ge
D
at
tb
mg
m4
pp
ms
E
be
se
gn

← Fig. S11.

Head morphology of *Siphonorhora* sp. (F8), sections based on µCT data. Arrows indicate articulation of the mandible. **(A)** Sagittal-section through midline of head. **(B)** Cross-section anterior of antennae. **(C)** Cross-section through head at level of antennae. **(D)** Transversal-section through mandibular base and tentorial complex. **(E)** Cross-section trough beak. **Scale:** A, D = 100 µm, B, C= 50 µm, E = 10 µm. **Abbreviations:** at = Antennae, be = beak, cly = clypeus, co = collum, eb = tentorial epipharyngeal bar, epc = epicranium, fro = frons, ge = gena, gn = gnathochilarium, la = labrum, m# = mandibular musculature, mc = mandibular cardo, mg = mandibular gnathal lobe, mgs = mandibular gnathal lobe sclerite, ms = mandibular stipes, p# = pharyngeal musculature, pc = pumping chamber, ph = pharynx, pp = tentorial posterior process, st = stylet, tb = tentorial transverse bar.

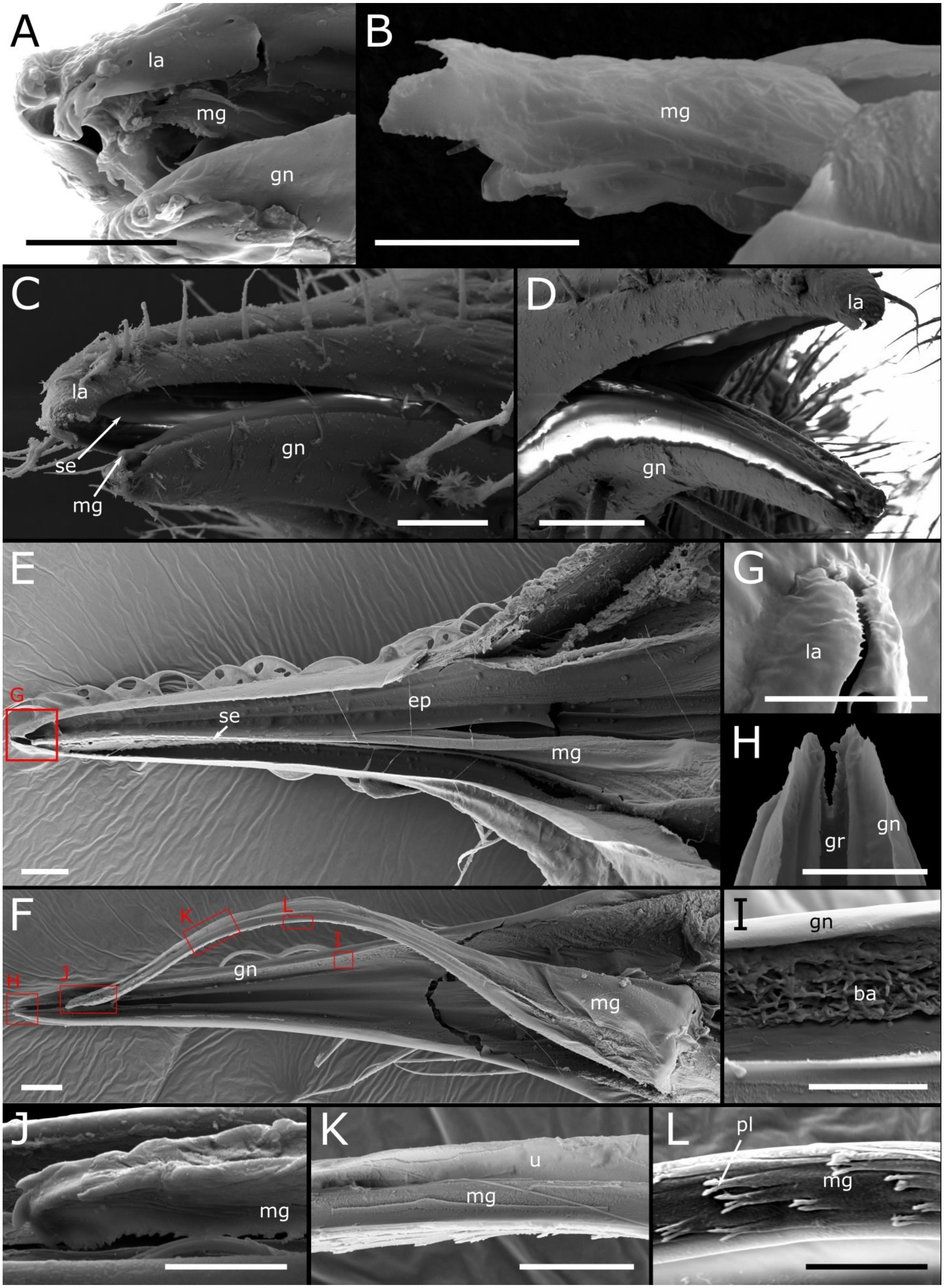

A
la
mg
gn
B
mg
C
la
se
gn
mg
D
la
gn
E
G
se
ep
mg
G
la
H
gr
gn
F
K
L
I
H
J
gn
mg
I
gn
ba
J
mg
K
u
mg
L
pl
mg

← **Fig. S12.**

Mandibular gnathal lobe of the Polyzoniida and Siphonophoridae, details, SEM. **(A, B)** *Rhinotus purpureus* (ZFMK-MYR10052). **(A)** Dilated gnathochilarium revealing the tips of the gnathal lobes. **(B)** Mandible gnathal lobe, ventral view. **(C, D)** *Siphonophora* sp. (ZFMK-MYR11177), with dilated gnathochilarium revealing the tips of the gnathal lobes. **(E – L)** *Siphonophora* cf. *zelandica* (ZFMK-MYR11173) **(E)** Cranium with right gnathal lobe, ventral view. **(F)** Gnathochilarium with left gnathal lobe, dorsal view. **(G)** Labrum, ventral view. **(H)** Apical tip of gnathochilarium, dorsal view. **(I)** Inner margin of gnathochilarium. **(J)** Apical tip of mandibular gnathal lobe. **(K)** Dorsal surface of mandibular gnathal lobe. **(L)** Outer surface of mandibular gnathal lobe. **Scale:** A, B, G – L = 10 µm, C – F = 20 µm. **Abbreviations:** ba = band of elongated cuticular fibers on gnathochilarium, ep = epipharynx, gn = gnathochilarium, gr = groove on inner surface of gnathochilarium, la = labrum, mg = mandibular gnathal lobe, pl = lamellae on the lateral surface of the gnathal lobe, se = median septum from epipharynx, u = dorsal u-shaped excavation of gnathal lobe.

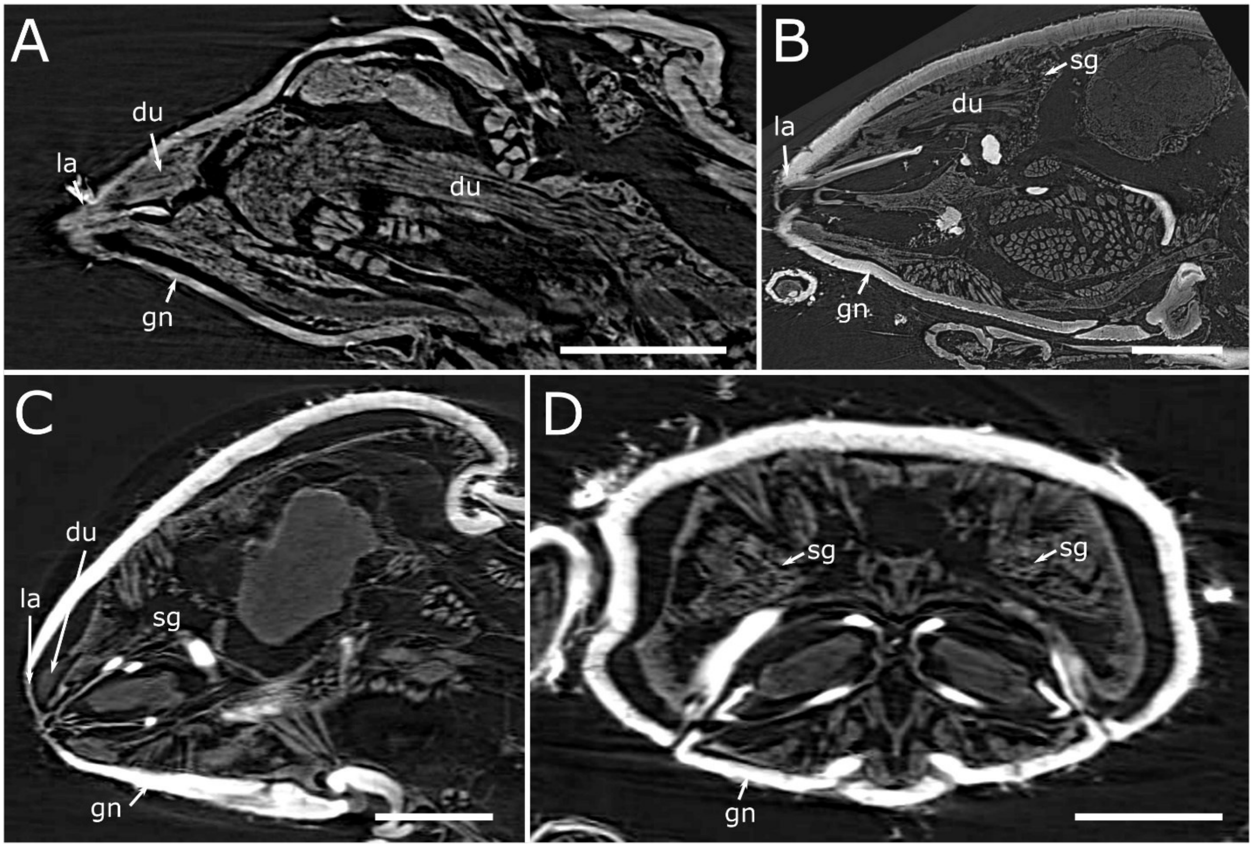

Fig. S13.

Salivary gland of colobognathan millipedes. **(A)** *Hirudicryptus canariensis* (Loksa, 1967) (ZFMK-MYR04780), sagittal section based on SR-µCT data. **(B)** Siphonorhinus sp. (F9), sagittal section based on SR-µCT data. **(C, D)** *Brachycybe lecontii*, SR-µCT data from (12). **(C)** Sagittal section. **(D)** Cross-section. **Scale:** A, C, D = 50 µm, B = 100 µm. **Abbreviations:** du = ducts of salivary gland, gn = gnathochilarium, la = labrum, sg = salivary gland.

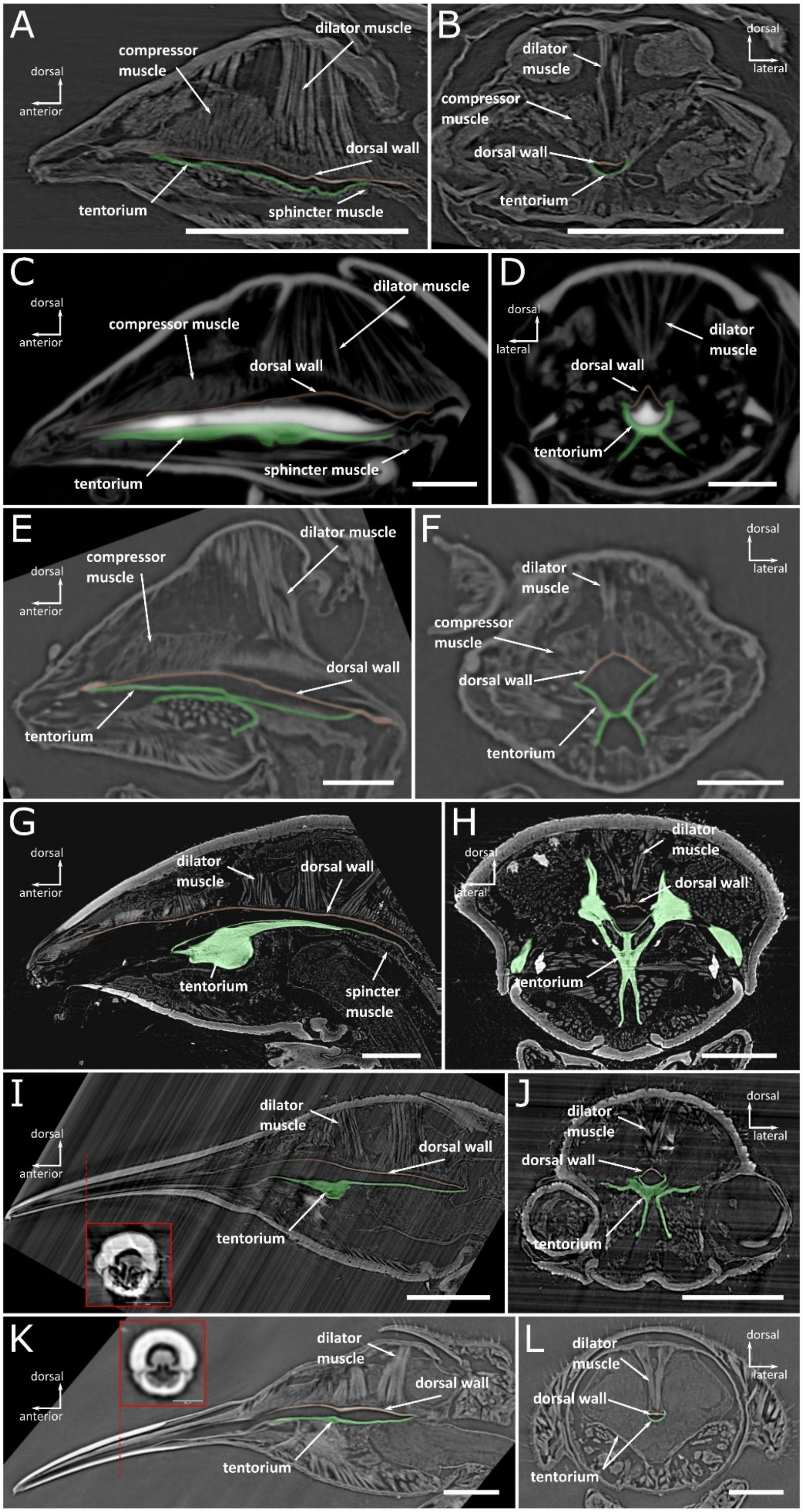
A
dilator muscle
compressor muscle
dorsal
anterior
dorsal wall
tentorium
sphincter muscle
B
dilator muscle
dorsal
lateral
compressor muscle
dorsal wall
tentorium
C
dorsal
anterior
compressor muscle
dilator muscle
dorsal wall
tentorium
sphincter muscle
D
dorsal
lateral
dilator muscle
dorsal wall
tentorium
E
dilator muscle
compressor muscle
dorsal
anterior
dorsal wall
tentorium
F
dorsal
lateral
dilator muscle
compressor muscle
dorsal wall
tentorium
G
dorsal
anterior
dilator muscle
dorsal wall
tentorium
spincter muscle
H
dilator muscle
dorsal
lateral
dorsal wall
tentorium
I
dorsal
anterior
dilator muscle
dorsal wall
tentorium
J
dorsal
lateral
dilator muscle
dorsal wall
tentorium
K
dorsal
anterior
dilator muscle
dorsal wall
tentorium
L
dilator muscle
dorsal
lateral
dorsal wall
tentorium

← Fig. S14.

Sucking pump in the Colobognatha, sections based on µCT and SR-µCT data. **(A, B)** *Hirudicryptus canariensis* (Loksa, 1967) (ZFMK-MYR04780). **(C, D)** *Rhinotus purpureus* (Pocock, 1894) (ZFMK-MYR11197) **(E, F)** *Hirudisoma roseum* (Victor, 1839) (ZFMK-MYR11195). **(G, H)** *Siphonorhinus* sp. (F9). **(I, J)** *Siphonophora* sp. (F8). **(K, L)** *Siphonophora* sp. (INDOSYMBIOSYS-CCDB26227-E04). **(A, C, E, G, I, K)** Sagittal sections. **(B, D, F, H, J, L)** Cross sections. **(I, K)** Detail of beak cross section in red box, arrow indicates position of cross section. **Scale** = 100 µm.

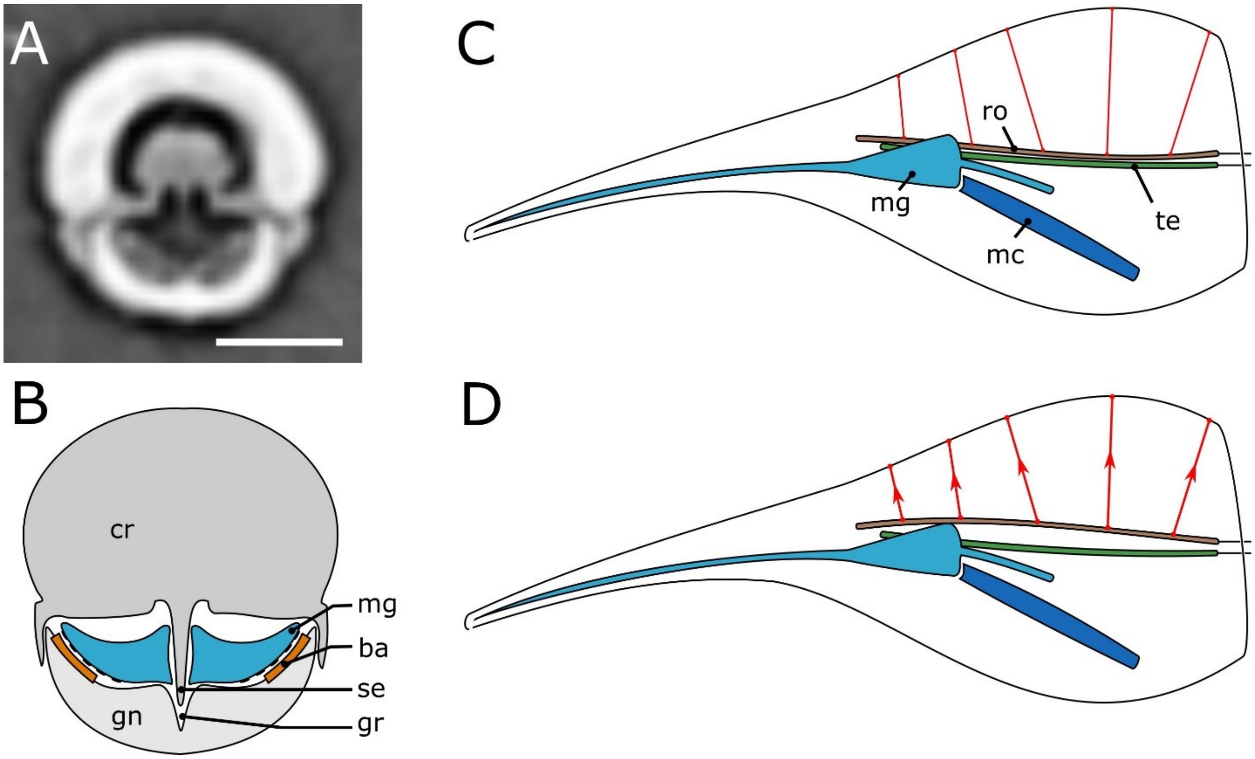

Fig. S15.
The potential feeding mechanism of the Siphonophoridae. **(A, B)** Cross-section through the beak of *Siphonophora* sp.**(A)** SR-µCT data (INDOSYMBIOSYS-CCDB26227-E04) **(B)**Schematic reppresentation. (**C, D)** Schematic sagittal section of head with relaxed **(C)** and contracted **(D)** pharyngeal dilator muscles. **Scale:** A = 20 µm. **Abbreviations:** ba = band of elongated cuticular fibers on gnathochilarium, cr = cranium, gn = gnathochilarium, gr = groove, mc = mandible cardo, mgl = mandible gnathal lobe, se = septum of beak.

Order	Family	Species	Sex	Voucher	Treatment	Method
Polyzoniida	Hirudisomatidae	*Hirudisoma roseum* (Victor, 1839)	f	ZFMK-MYR11195	Bouin, Iodine staining, CPD	SR-µCT (DESY)
Polyzoniida	Hirudisomatidae	*Hirudisoma roseum* (Victor, 1839)	f	ZFMK-MYR7255	96% EtOH, CPD	SEM
Polyzoniida	Polyzoniidae	*Polyzonium germanicum* Brandt, 1837	f	ZFMK-MYR11196	Bouin, Iodine staining, CPD	SR-µCT (DESY)
Polyzoniida	Polyzoniidae	*Polyzonium germanicum* Brandt, 1837	f	ZFMK-MYR9888	96% EtOH, CPD	SEM
Polyzoniida	Polyzoniidae	*Polyzonium germanicum* Brandt, 1837	m	ZFMK-MYR9888	96% EtOH, CPD	SEM
Polyzoniida	Polyzoniidae	*Polyzonium germanicum* Brandt, 1837	m	ZFMK-MYR9888	96% EtOH, Iodine	µCT
Polyzoniida	Siphonotidae	*Rhinotus purpureus* (Pocock, 1894)	f	ZFMK-MYR11197	Bouin, Iodine staining, CPD	SR-µCT (PSI)
Polyzoniida	Siphonotidae	*Rhinotus purpureus* (Pocock, 1894)	f	ZFMK-MYR10052	96% EtOH, CPD	SEM
Polyzoniida	Siphonotidae	*Rhinotus purpureus* (Pocock, 1894)	m	ZFMK-MYR10052	96% EtOH, CPD	SEM
Polyzoniida	Siphonotidae	*Siphonethus* sp.	f	ZFMK-MYR11180	96% EtOH, CPD	SEM
Siphonocryptida	Siphonocryptidae	*Hirudicryptus canariensis* (Loksa, 1967)	f	ZFMK-MYR04780	96% EtOH, CPD	SR-µCT (Spring8)
Siphonocryptida	Siphonocryptidae	*Hirudicryptus canariensis* (Loksa, 1967)	f	ZFMK-HIST00002	Bouin	Histology
Siphonocryptida	Siphonocryptidae	*Hirudicryptus canariensis* (Loksa, 1967)	m	ZFMK-HIST00003	Bouin	Histology
Siphonocryptida	Siphonocryptidae	*Hirudicryptus canariensis* (Loksa, 1967)	f	ZFMK-MYR08906	96% EtOH, CPD	SEM
Siphonocryptida	Siphonocryptidae	*Hirudicryptus canariensis* (Loksa, 1967)	m	ZFMK-MYR08906	96% EtOH, CPD	SEM
Siphonophorida	Siphonophoridae	*Siphonophora* sp	f	F8	96% EtOH, CPD	SR-µCT (Spring8)
Siphonophorida	Siphonophoridae	*Siphonophora* sp	f	INDOSYMBIOSYS-CCDB26227-E04	96% EtOH, CPD	SR-µCT (DESY)
Siphonophorida	Siphonophoridae	*Siphonophora* cf. *zelandica*	f	ZFMK-MYR11173	96% EtOH, CPD	SEM
Siphonophorida	Siphonophoridae	*Siphonophora* sp.	f	ZFMK-MYR11177	96% EtOH, CPD	SEM
Siphonophorida	Siphonophoridae	*Siphonophora* cf. *zelandica*	f	NZAC03038958	96% EtOH, CPD	SEM
Siphonophorida	Siphonorhinidae	*Siphonorhinus* sp.	f	F9	96% EtOH, CPD	SR-µCT (Spring8)
Siphonophorida	Siphonorhinidae	*Siphonorhinus* sp.	f	MHNG	70% EtOH, CPD	SEM

Table S1.

Taxon sampling and treatment of specimens. CPD = critical point dried, EtOH = ethanol, f = female µCT = micro-computed tomography, m = male; SEM = scanning electron microscopy, SR-µCT = Synchrotron micro-computed tomography. Pixel size is given after binning.

	Hirudisoma roseum	*Polyzonium germanicum*	*Rhinotus purpureus*	*Hirudicryptus canariensis*	*Siphonophora sp. (Spring8)*	*Siphonophora sp. (DESY)*	*Siphonorhinus sp. (Spring8)*
**Head capsule length (µm)**	510	330	350	162	560	815	625
**Head width (measured at widest) (µm)**	390	295	180	128	270	321	504
**Mandibular cardo length (µm)**	60	30	30	-	26	-	66
**Mandibular cardo width (µm)**	15	20	12	-	14	-	31
**Mandibular stipes length (µm)**	270	150	220	-	130	-	215
**Mandibular stipes width (µm)**	25	20	10	-	16	-	26
**Gnathal lobe length (µm)**	60	55	30	-	344	-	276
**Gnathal lobe sclerite length (µm)**	270	150	210	-	75	-	217
**Stipes length/cardo length**	4.5	5	7.33333	-	5.826	-	3.25
**Length sucking pump (µm)**	440	207	250	128	239	-	424
**Diameter sucking pump (µm)**	90	32	32	16	35	-	42
**Volume sucking pump (µl)**	0.0009256	0.000307	0.00005703	0.000003679	0.000007138	-	0.0002837

Table S2.

Measurements of skeletal elements of the Polyzoniida and Siphonocryptida. All Measurements in µm.

**Muscle**	**Origin**	**Insertion**	***Hirudisoma roseum***	***Polyzonium germanicum***	***Rhinotus purpureus***	***Hirudicryptus canariensis***	***Siphonorhinus* sp.**	***Siphonophra sp.***	***Brachycybe lecontii***	***Dolistenus sp.***
**a1**	Cranium, median on posterior margin	Antennal base, posterior margin	+	?	+	+	+	+	+	+
**a2**	Tentorium, posterior process	Antennal base, posterior margin	+	?	+	+	+	+	+	+
**a3**	Tentorium, posterior process	Antennal base, anterior lateral margin	+	?	+	+	+	+	+	+
**a4**	Tentorium, posterior process	Antennal base, anetrior median margin	+	?	+	+	+	+	+	+
**a5**	Tentorium, posterior process	Antennal base, posterior lateral margin	-	-	-	-	+	+	-	-
**g1**	Hypopharyngeal lateral sclerite/tentorium	Gnathochilarium (lamella lingualis/anterior)	+	+	+	+	+	+	+	+
**g2**	Hypopharyngeal lateral sclerite/tentorium	Gnathochilarium (stipes/lateral)	+	+	+	+	+	+	+	+
**g3**	Hypopharyngeal lateral sclerite/tentorium	Gnathcohilarium (mentum/anterior mesally)	+	+	+	+	+	+	+	+
**g4**	Hypopharynx/tentorium	Gnathochilarium (mentum/middle, mesally)	+	+	+	+	+	+	+	+
**g5**	Tentorium, posterior process	Gnathochilarium (Stipes/posterior lateral)	+	+	+	+	+	+	+	+
**g6**	Cranium, posterior of mandibular base (cardo)	Gnathochilarium (stipes)	-	-	-	-	-	-	+	+
**g7**	Gnthochilarium, posterior margin	Gnathochilarium (lateral)	+	+	+	?	+	-	-	-
**g8**	Tentorium, posterior process	Gnathochilarium (posterior margin)	+	+	+	?	+	+	-	-
**m1**	Cranium (posterior margin/posocciital process)	Gnathal lobe sclerite	+	+	+	+	+	+	+	+
**m2**	Cranium (posterior margin/posocciital process)	Mandibular stipes	+	+	+	+	+	+	+	+
**m3**	Tentorium, posterior process	Mandibular stipes	+	+	+	+	+	+	-	-
**m4**	Tentorium, posterior process	Mandibular stipes	+	+	+	+	+	+	+ (m3)	+ (m3)
**m5**	Tentorium, posterior process	Mandibular stipes	+	+	+	+	+	+	+ (m4)	+ (m4)
**p1**	Cranium, frons	Pharynx, dorsally/Dorsal wall of sucking pump	+	+	+	+	+	+	+	+
**p2**	Cranium, frons	Pharynx, dorsally/Dorsal wall of sucking pump	+	+	+	+	+	+	+	+
**p3**	Cranium, frons/epicranium	Pharynx, dorsally/Dorsal wall of sucking pump	+	+	+	+	+	+	+	+
**p4**	Cranium, posterior margin	Pharynx, dorsally	-	-	-	-	-	-	+	+
**p5**	Tentorium, posterior process	Pharynx, ventral	-	-	-	-	+	-	+	+
**p6**	Transverse mandibular tendon	Pharynx, ventral	-	-	-	-	-	-	+	+
**p7**	Gula	Pharynx ventral	-	-	-	-	+	+	+	+
**s1**	Around opening of foregut		+	+	+	+	+	-	-	-
**c1**	Across dorsal wall of sucking pump		+	+	+	+	-	-	-	-
**t1**	Cranium, clypeus/frons, anterior	Tentorium (epipharyngeal bar)	+	+	+	+	+	+	+	+

**t2**	Cranium, frons, posterior	Tentorium (epipharyngeal bar)	+	+	+	+	+	+	+	+
**t3**	Cranium (posterior margin/posocciital process)	Tentorium posterior process	-	-	-	-	+	+	+	+
**t4**	Gula, anterior margin	Tentorium, posterior process	-	-	-	-	-	-	+	+
**t5**	Cranium, frons	Tentorium, dorsal	+	+	+	+	-	-	-	-
**e1**	Cranium, clypeus	Epipharynx	-	-	-	-	+	+	-	-
**e2**	Cranium, clypeus	Epipharynx	-	-	-	-	+	+	+	+
**e3**	Cranium, clypeus	Epipharynx	-	-	-	-	+	+	+	+

Table S3.

Cephalic musculature of the Colobognatha. The Platydemida *Brachycybe lecontii* and *Dolistenus* sp. have been studied by Moritz et al. (22). + = present; - = absent; ? = unknown.

Taxa	Taxa	Structure of the pumping chamber	Musculature of the pumping chamber	"valves"	Comments	References
Arachnida	Acari	Pharyngeal pump	Dorsal and ventrolateral dilator muscles; surrounded by constrictor muscles	-	-	(6)
Arachnida	Amblypygi	Precerebral pharyngeal pump	Dorsal + lateral pharyngeal dilator muscles; constrictor muscle	-	-	(81)
Arachnida	Arnaeae	Proventricular pump/"sucking stomach", Y-shaped	Dorsal + ventrolateral dilator muscles; compressor muscles	-	-	(6, 82)
Arachnida	Opiliones	Pharyngeal pump	Dorsal, lateral + ventral dilator muscles; surrounded by circular compressor muscles	-	-	(6)
Arachnida	Palpigradi	"Sucking stomach" formed by esophagus	Dorsal + ventral dilator muscles	-	-	(6)
Arachnida	Pseudoscorpiones	Pharyngeal pump; X-shaped	Dorsal + lateral dilator muscles; surrounded by constrictor muscles	-	-	(6)
Arachnida	Ricinulei	Precerebral pump; ventral sclerotized floor; flexible roof	Dorsal dilator muscles; no compressor muscles	-	Additionally, postcerebral sucking pump formed by esophagus, showing same pattern as sucking stomach of other arachnids	(40)
Arachnida	Scorpiones	Precerebral pharyngeal pump, triangular	Dorsal and lateral dilator muscles; dorsal and lateral constrictor muscles	-	-	(83)
Arachnida	Solifugae	Precerebral pharyngeal pump, Y-shaped	Dorsal and lateral dilator muscles; dorsal and lateral constrictor muscles	-	-	(6, 84)
Arachnida	Uropygi	Pharyngeal pump	Dorsal + lateral dilator muscles;	.	-	(85)

			constrictor muscles			
Arachnida		Pharyngeal pump	Dorsal and (ventro-)lateral dilator muscles; constrictor musles	-	-	(6)
Chilopoda	Geophilomorpha	Pharynx; X-/pipette-shaped, thickened walls	Lateral dilator muscles; constrictor muscles	-	Structure and function largely unknown	(31, 32)
Crustacea	Branchiura (Argulidae)	Esophagus	Dorsal, lateral + ventral dilator muscles; Constrictor and longitudinal muscles surrounding esophagus	Oral opening can be closed	Emptying of pump by contraction of constrictor and longitudinal muscles	(86)
Crustacea	Isopoda (Aegidae, Cymothoidae)	Esophagus (can be widened actively at two locations)	Dorsal, lateral + ventral(?) dilator muscles; compressor around esophagus	Anterior sphincter muscle; esophagus closed posteriorly by chitinized plate	-	(7)
Diplopoda	Polyzoniida	Preoralchamber/pharynx; floor strong, sclerotized(?) supported by tentorial complex; Roof thin	Dorsal dilator muscles; dorsal compressor muscles	Posterior sphincter muscle	Emptying of pump by compressor muscles	This study
Diplopoda	Siphonocryptida	Preoralchamber/pharynx, floor strong, sclerotized(?) supported by tentorial complex; Roof thin	Dorsal dilator muscles; dorsal compressor muscles	Posterior sphincter muscle	Emptying of pump by compressor muscles	This study
Diplopoda	Siphonophora (Siphonophoridae)	Preoralchamber/pharynx; floor strong, sclerotized(?) supported by tentorial complex; Roof thin	Dorsal dilator muscles; no compressor muscles	-	Emptying of pump by constrictor muscles	This study
Diplopoda	Siphonophora (Siphonorhinidae)	Preoralchamber/pharynx; floor strong, sclerotized(?) supported by tentorial complex; Roof thin	Dorsal dilator muscles; no compressor muscles	Posterior sphincter muscle	Emptying of pump by constrictor muscles	This study
Hexapoda	Anoplura/Phthiraptera	Cibarial pump; solid floor; flexible roof	Dorsal dilator muscles; no compressor muscles	-	Two pumps, 1 cibarial (discussed here). 1 pharyngeal	(38)
Hexapoda	Coleoptera (Elateridae larvae)	Cibarial pump; floor with thicker intima, roof flexible	Dorsal dilator muscles; dorsal compressor muscles in transverse orientation	-	Emptying of pump by compressor muscles	(48, 49)

Hexapoda	Coleoptera (Some Caraboidea)	Cibarial pump; floor sclerotized, supported by suspensorium	Dorsal dilator muscles; dorsal compressor muscles in transverse orientation	-	Emptying of pump by compressor muscles	(42)
Hexapoda	Collembola	Pharyngeal pump	Dorsal + ventral dilator muscles; circumpharyngeal constrictor muscles	Anteriorly closed by labral depressors	Emptying of pump by constrictor muscles	(29)
Hexapoda	Diptera	Cibarial pump; solid floor/sclerotized ventral/posterior wall; flexible roof (dorsal/anterior wall)	Dorsal dilator muscles; no compressor muscles	Posteriorly closed by dorsal plate	Emptying of pump by elastic retraction of dorsal wall; additional pharyngeal pump	(8, 38, 45, 87, 88)
Hexapoda	Hemiptera (Auchenorrhyncha)	Cibarial pump; ventral wall strongly sclerotized, deep oval basin; flexible dorsal wall	Dorsal dilator muscles; no compressor muscles	-	Emptying of pump by elastic retraction of piston (dorsal wall)	(8, 38, 89, 90)
Hexapoda	Hemiptera (Heteroptera)	Cibarial pump; V- or U- shaped sclerotized floor; dorsal wall, flexible piston	Dorsal dilator muscles; no compressor muscles	Precibarial valve;	Emptying of pump by elastic retraction of piston (dorsal wall)	(38, 44, 54, 89–93)
Hexapoda	Hemiptera (Sternorrhyncha)	Cibarial pump; ventral wall thick; dorsal wall thinner and flexible	Dorsal dilator muscles; no compressor muscles	Maybe pharynx protuberences acting as valves	Emptying of pump by elastic retraction of piston (dorsal wall)	(8, 33, 34, 89, 90, 94–96)
Hexapoda	Hymenoptera (Xyloidea, Apoidea)	Cibarial pump; ventral sclerotized floor; flexible roof	Ddorsal dilator muscles; dorsal compressor muscles in longitudinal? and/or circular? orientation	-	Emptying of pump by compressor muscles	(36, 37, 46, 47, 97)
Hexapoda	Lepidoptera	Cibarial pump; ventral wall formed by hypopharynx, strongly sclerotized, with median groove; dorsal wall weakly sclerotized, flexible;	Dorsal dilator muscles; dorsal compressor muscles in layer above dorsal wall	Oral valve (or sphincter muscle/cibarial closer) anteriorly; Posterior sphincter muscles	Emptying of pump by compressor muscles	(8, 11, 35, 41, 43, 98)
Hexapoda	Mecoptera	Cibarial pump; ventral sclerotized floor	Dorsal dilator muscles; no compressor muscles	-	Present in some taxa	(36, 97, 99)
Hexapoda	Protura	Pharyngeal pump	Dorsal + lateral dilator muscles; circumpharyngeal constrictor	-	Emptying of pump by constrictor muscles	(30)

			muscles			
Hexapoda	Psocodea	Cibarial pump	Dorsal dilator muscles; no compressor muscles	-		(38)
Hexapoda	Siphonaptera	Cibarial pump; ventral wall formed by sclerotized sitophore; dorsal wall thin and flexible	Dorsal dilator muscles; no compressor muscles	Valves seperate cibarial pump from pre- and postpharyngeal pumps	Emptying of pump by compressor muscles; 3 pumps: 1 prepharyngeal, 1 cibarial, 1 postpharyngeal pump	(8, 38)
Hexapoda	Thysanoptera	Cibarial pump; ventral wall sclerotized; dorsal wall flexible	Dorsal dilator muscles; no compressor muscles	-	Emptying of pump by elastic retraction of piston (dorsal wall)	(11, 38, 39)
Hexapoda	Trichoptera	Cibarial pump	Dorsal dilator muscles; dorsal compressor muscles	-		(100)
Onychophora	Onychophora	Triradiate sucking pharynx	Radial and circular muscles	-	Peristaltic movement	(4)
Tardigrada	Tardigrada	Triradiate sucking pharynx	Myoepithel	-	Peristaltic movement	(4)

Table S4.

Sucking pumps in Arthropod. - = not reported/unknow

REFERENCES AND NOTES

1. O. F. Cook, H. F. Loomis, Millipeds of the order Colobognatha, with descriptions of six new genera and type species, from Arizona and California. *Proc. U.S. Natl. Museum* **72**, 1–26 (1928).

2. J. F. Brandt, Ueber saugende Myriapoden (*Colobognatha*). *Isis von Oken* **13**, 704 (1834).

3. S. M. Manton, The evolution of arthropodan locomotory mechanisms. Part 71. Functional requirements and body design in Colobognatha (Diplopoda), together with a comparative account of diplopod burrowing techniques, trunk musculature and segmentation. *Zool. J. Linn. Soc.* **44**, 383–462 (1961).

4. C. Nielsen, The triradiate sucking pharynx in animal phylogeny. *Invertebr. Biol.* **132**, 1–13 (2013).

5. W. Hüther, Zur Ernährung der Pauropoden. *Naturwissenschaften* **46**, 563–564 (1959).

6. R. E. Snodgrass, The feeding organs of Arachnida, including mites and ticks. *Smithson. Misc. Collect.* **110**, 1–93 (1948).

7. K. Günther, Bau und funktion der mundwerkzeuge bei crustaceen aus der familie der Cymothoidae (Isopoda). *Zoomorphology* **23**, 1–79 (1931).

8. H. W. Krenn, Fluid-feeding mouthparts, in *Insect Mouthparts: Form, Function, Development and Performance*, H. W. Krenn, Ed. (Springer, 2019), pp. 47–99.

9. P. H. Adler, R. G. Foottit, Introduction, in *Insect Biodiversity* (Wiley, 2017), pp. 1–7.

10. K. G. Kornev, P. H. Adler, Physical determinants of fluid-feeding in insects, in *Insect Mouthparts: Form, Function, Development and Performance*, H. W. Krenn, Ed. (Springer, 2019), pp. 263–314.

11. R. E. Snodgrass, *Principles of Insect Morphology* (McGraw-Hill Book, 1935).

12. K. G. Kornev, A. A. Salamatin, P. H. Adler, C. E. Beard, Structural and physical determinants of the proboscis–sucking pump complex in the evolution of fluid-feeding insects. *Sci. Rep.* **7**, 6582 (2017).

13. S. M. Manton, Mandibular mechanisms and evolution of arthropods. *Philos. Trans. R. Soc. Lond. B Biol. Sci.* **247**, 1–183 (1964).

14. R. L. Hoffman, Diplopoda, in *Synopsis and Classification of Living Organisms*, S. P. Parker, Ed. (McGraw-Hill, 1982), pp. 689–719.

15. M. Koch, Diplopoda—General morphology, in *Treatise on Zoology - Anatomy, Taxonomy, Biology. The Myriapoda, Volume 2*, A. Minell, Ed. (Brill, 2015), pp. 7–67.

16. A. M. Macias, P. E. Marek, E. M. Morrissey, M. S. Brewer, D. P. G. Short, C. M. Stauder, K. L. Wickert, M. C. Berger, A. M. Metheny, J. E. Stajich, G. Boyce, R. V. M. Rio, D. G. Panaccione, V. Wong, T. H. Jones, M. T. Kasson, Diversity and function of fungi associated with the fungivorous millipede, *Brachycybe lecontii*. *Fungal Ecol.* **41**, 187–197 (2019).

17. H. Hauser, K. Voigtländer, *Doppelfüßer (Diplopoda) Deutschlands: Verhalten, Ökologie, Verbreitung, Lebendbestimmung* (Deutscher Jugendbund für Naturbeobachtung, 2019).

18. V. Wong, D. Hennen, A. Macias, M. Brewer, M. Kasson, P. Marek, Natural history of the social millipede *Brachycybe lecontii* Wood, 1864. *Biodivers. Data J.* **8**, e50770 (2020).

19. P. E. Marek, W. A. Shear, J. E. Bond, A redescription of the leggiest animal, the millipede *Illacme plenipes*, with notes on its natural history and biogeography (Diplopoda, Siphonophorida, Siphonorhinidae). *ZooKeys* **2012**, 77–112 (2012).

20. H. Enghoff, S. Golovatch, M. Short, P. Stoev, T. Wesener, Diplopoda—Taxonomic overview, in *Treatise on Zoology - Anatomy, Taxonomy, Biology. The Myriapoda, Volume 2*, A. Minell, Ed. (Brill, 2015), pp. 363–453.

21. A. Blanke, T. Wesener, Revival of forgotten characters and modern imaging techniques help to produce a robust phylogeny of the Diplopoda (Arthropoda, Myriapoda). *Arthropod Struct. Dev.* **43**, 63–75 (2014).

22. L. Moritz, A. Blanke, J. U. Hammel, T. Wesener, First steps toward suctorial feeding in millipedes: Comparative morphology of the head of the Platydesmida (Diplopoda: Colobognatha). *Invertebr. Biol.* **140**, e12312 (2021).

23. J. Jurin, II. An account of some experiments shown before the Royal Society; with an enquiry into the cause of the ascent and suspension of water in capillary tubes. *Philos. Trans. R. Soc. Lond.* **30**, 739–747 (1719).

24. K. G. Kornev, D. Monaenkova, C. Yore, C. Klipowics, K. Edmond, V. Sa, T. Andrukh, Butterfly proboscis as a biomicrofluidic system, in *American Physical Society, 62nd Annual Meeting of the APS Division of Fluid Dynamics* (2009).

25. D. Monaenkova, M. S. Lehnert, T. Andrukh, C. E. Beard, B. Rubin, A. Tokarev, W.-K. Lee, P. H. Adler, K. G. Kornev, Butterfly proboscis: Combining a drinking straw with a nanosponge facilitated diversification of feeding habits. *J. R. Soc. Interface* **9**, 720–726 (2012).

26. D. B. Parker, Positive displacement pumps - performance and application. in *Proceedings of the 11th International Pump Users Symposium* (Turbomachinery Laboratories, Department of Mechanical Engineering, Texas A&M University, 1994).

27. D. Bach, F. Schmich, T. Masselter, T. Speck, A review of selected pumping systems in nature and engineering—Potential biomimetic concepts for improving displacement pumps and pulsation damping. *Bioinspir. Biomim.* **10**, 051001 (2015).

28. W. H. Fahrenbach, C. P. Arango, Microscopic anatomy of Pycnogonida: II. Digestive system. III. Excretory system. *J. Morphol.* **268**, 917–935 (2007).

29. H. Wolter, Vergleichende Untersuchungen zur Anatomie und Funktionsmorphologie der stechend-saugenden Mundwerkzeuge der Collembolen. *Zool. Jahrb. Anat.* **81**, 27–100 (1963).

30. J. François, R. Dallai, W. Y. Yin, Cephalic anatomy of *Sinentomon erythranum* Yin (Protura: Sinentomidae). *Int. J. Insect Morphol. Embryol.* **21**, 199–213 (1992).

31. S. M. Manton, The evolution of arthropodan locomotory mechanisms. Part 8. Functional requirements and body design in Chilopoda, together with a comparative account of their skeleto-muscular systems and an appendix on a comparison between burrowing forces of annelids and chilopods and its bearing upon the evolution of the arthropodan haemocoel. *Zool. J. Linn. Soc.* **45**, 251–484 (1965).

32. M. Koch, G. D. Edgecombe, The preoral chamber in geophilomorph centipedes: Comparative morphology, phylogeny, and the evolution of centipede feeding structures. *Zool. J. Linn. Soc.* **165**, 1–62 (2012).

33. A. R. Forbes, The morphology, histology, and fine structure of the gut of the green peach aphid, *Myzus persicae* (Sulzer) (Homoptera: Aphididae). *Mem. Entomol. Soc. Can.* **96**, 5–74 (1964).

34. A. R. Forbes, The mouthparts and feeding mechanism of aphids, in *Aphids As Virus Vectors*, K. F. Harris, K. Maramorosch, Eds. (Academic Press, 1977), pp. 83–103.

35. S. H. Eberhard, H. W. Krenn, Anatomy of the oral valve in nymphalid butterflies and a functional model for fluid uptake in Lepidoptera. *Zool. Anz. J. Comp. Zool.* **243**, 305–312 (2005).

36. L. Vilhelmsen, The preoral cavity of lower Hymenoptera (Insecta): Comparative morphology and phylogenetic significance. *Zool. Scr.* **25**, 143–170 (1996).

37. J. A.-S. Bauder, F. Karolyi, Superlong proboscises as co-adaptations to flowers, in *Insect Mouthparts: Form, Function, Development and Performance*, H. W. Krenn, Ed. (Springer, 2019), pp. 479–527.

38. R. E. Snodgrass, The feeding apparatus of biting and sucking insects affecting man and animals. *Smithson. Misc. Collect.* **104**, 1–113 (1944).

39. D. E. Ullman, D. M. Westcot, W. B. Hunter, R. F. L. Mau, Internal anatomy and morphology of *Frankliniella occidentalis* (Pergande) (Thysanoptera: Thripidae) with special reference to interactions between thrips and tomato spotted wilt virus. *Int. J. Insect Morphol. Embryol.* **18**, 289–310 (1989).

40. G. Talarico, E. Lipke, G. Alberti, Gross morphology, histology, and ultrastructure of the alimentary system of Ricinulei (Arachnida) with emphasis on functional and phylogenetic implications. *J. Morphol.* **272**, 89–117 (2011).

41. J. B. Schmitt, The feeding mechanism of adult Lepidoptera. *Smithson. Misc. Collect.* **97**, 1–27 (1938).

42. M. E. G. Evans, T. G. Forsythe, Feeding mechanisms, and their variation in form, of some adult ground-beetles (Coleoptera: Caraboidea). *J. Zool.* **206**, 113–143 (1985).

43. N. T. Davis, J. G. Hildebrand, Neuroanatomy of the sucking pump of the moth, *Manduca sexta* (Sphingidae, Lepidoptera). *Arthropod Struct. Dev.* **35**, 15–33 (2006).

44. H. C. Bennet-Clark, Negative pressures produced in the pharyngeal pump of the blood-sucking bug, *Rhodnius Prolixus*. *J. Exp. Biol.* **40**, 223–229 (1963).

45. M. J. Rice, Function of resilin in tsetse fly feeding mechanism. *Nature* **228**, 1337–1338 (1970).

46. R. E. Snodgrass, The skeleto-muscular mechanisms of the honey bee. *Smithson. Misc. Collect.* **103**, 1–120 (1942).

47. R. G. Beutel, L. Vilhelmsen, Head anatomy of Xyelidae (Hexapoda: Hymenoptera) and phylogenetic implications. *Org. Divers. Evol.* **7**, 207–230 (2007).

48. D. C. Eidt, Anatomy and histology of the full-grown larva of *Ctenicera aeripennis destructor* (brown) (Coleoptera: Elateridae). *Can. J. Zool.* **36**, 317–361 (1958).

49. D. C. Eidt, Mode of feeding of the larva of *Ctenicera aeripennis destructor* (Brown) (Coleoptera: Elateridae). *Can. Entomol.* **91**, 97–101 (1959).

50. P. E. Marek, J. K. Krejca, W. A. Shear, A new species of *Illacme* Cook & Loomis, 1928 from Sequoia National Park, California, with a world catalog of the Siphonorhinidae (Diplopoda, Siphonophorida). *ZooKeys* **626**, 1–43 (2016).

51. H. Fechter, Anatomie und Funktion der Kopfmuskulatur von *Cylindroiulus teutonicus*. *Zool. Jahrbuecher Abt. Fuer Anat. Ontog. Tiere.* **79**, 479–528 (1961).

52. H. M. Wilson, Muscular anatomy of the millipede *Phyllogonostreptus nigrolabiatus* (Diplopoda: Spirostreptida) and its bearing on the millipede "thorax". *J. Morphol.* **251**, 256–275 (2002).

53. B. Naumann, H. S. Reip, N. Akkari, D. Neubert, J. U. Hammel, Inside the head of a cybertype – three-dimensional reconstruction of the head muscles of *Ommatoiulus avatar* (Diplopoda: Juliformia: Julidae) reveals insights into the feeding movements of Juliformia. *Zool. J. Linn. Soc.* **188**, 954–975 (2020).

54. A. Blanke, The early evolution of biting-chewing performance in Hexapoda, in *Insect Mouthparts: Form, Function, Development and Performance*, H. W. Krenn, Ed. (Springer, 2019), pp. 175–202.

55. E. S. El-Hifnawi, G. Seifert, Speicheldrüsen der Diplopoden. I. Topographie und Histologie der Speicheldrüsen von *Polyxenus lagurus*, *Craspedosoma rawlinsii* und *Schizophyllum sabulosum*. *Z. Morphol. Okologie Tiere.* **74**, 323–348 (1973).

56. F. S. Nunez, C. S. Crawford, Digestive enzymes of the desert millipede *Orthoporus ornatus* (Girard) (Diplopoda: Spirostreptidae). *Comp. Biochem. Physiol. A Physiol.* **55**, 141–145 (1976).

57. J. Lewis, Notes on the biology of some common millipedes of the Gunung Mulu National Park, Sarawak, Borneo. *Sarawak Mus. J.* **3354**, 179–185 (1984).

58. É. Lorenceau, D. Quéré, Drops on a conical wire. *J. Fluid Mech.* **510**, 29–45 (2004).

59. Y.-E. Liang, H.-K. Tsao, Y.-J. Sheng, Drops on hydrophilic conical fibers: Gravity effect and coexistent states. *Langmuir* **31**, 1704–1710 (2015).

60. M. Hilgert, N. Akkari, C. Rahmadi, T. Wesener, The Myriapoda of Halimun-Salak National Park (Java, Indonesia): Overview and faunal composition. *Biodivers. Data J.* **7**, e32218 (2019).

61. T. Wesener, L. Moritz, Checklist of the Myriapoda in Cretaceous Burmese amber and a correction of the Myriapoda identified by Zhang (2017). *Check List.* **14**, 1131–1140 (2018).

62. X. Jiang, W. A. Shear, D. A. Hennen, H. Chen, Z. Xie, One hundred million years of stasis: *Siphonophora hui* sp. nov., the first Mesozoic sucking millipede (Diplopoda: Siphonophorida) from mid-Cretaceous Burmese amber. *Cretac. Res.* **97**, 34–39 (2019).

63. I. Greving, F. Wilde, M. Ogurreck, J. Herzen, J. U. Hammel, A. Hipp, F. Friedrich, L. Lottermoser, T. Dose, H. Burmester, M. Müller, F. Beckmann, P05 imaging beamline at PETRA III: First results, in *Developments in X-Ray Tomography IX* (International Society for Optics and Photonics, 2014), vol. 9212, p. 92120O.

64. A. Haibel, M. Ogurreck, F. Beckmann, T. Dose, F. Wilde, J. Herzen, M. Müller, A. Schreyer, V. Nazmov, M. Simon, A. Last, J. Mohr, Micro- and nano-tomography at the GKSS Imaging Beamline at PETRA III, in *Developments in X-Ray Tomography VII* (International Society for Optics and Photonics, 2010), vol. 7804, p. 78040B.

65. F. Wilde, M. Ogurreck, I. Greving, J. U. Hammel, F. Beckmann, A. Hipp, L. Lottermoser, I. Khokhriakov, P. Lytaev, T. Dose, H. Burmester, M. Müller, A. Schreyer, Micro-CT at the imaging beamline P05 at PETRA III. *AIP Conf. Proc.* **1741**, 030035 (2016).

66. M. Stampanoni, A. Groso, A. Isenegger, G. Mikuljan, Q. Chen, A. Bertrand, S. Henein, R. Betemps, U. Frommherz, P. Böhler, D. Meister, M. Lange, R. Abela, Trends in synchrotron-based tomographic imaging: The SLS experience, in *Developments in X-Ray Tomography V* (International Society for Optics and Photonics, 2006), vol. 6318, p. 63180M.

67. K. Uesugi, M. Hoshino, A. Takeuchi, Y. Suzuki, N. Yagi, Development of fast and high throughput tomography using CMOS image detector at SPring-8, in *Developments in X-Ray Tomography VIII* (International Society for Optics and Photonics, 2012), vol. 8506, p. 85060I.

68. J. Schindelin, I. Arganda-Carreras, E. Frise, V. Kaynig, M. Longair, T. Pietzsch, S. Preibisch, C. Rueden, S. Saalfeld, B. Schmid, J.-Y. Tinevez, D. J. White, V. Hartenstein, K. Eliceiri, P. Tomancak, A. Cardona, Fiji: An open-source platform for biological-image analysis. *Nat. Methods* **9**, 676–682 (2012).

69. P. A. Yushkevich, Y. Gao, G. Gerig, ITK-SNAP: An interactive tool for semi-automatic segmentation of multi-modality biomedical images, in *2016 38th Annual International Conference of the IEEE Engineering in Medicine and Biology Society (EMBC)* (2016), pp. 3342–3345.

70. P. Cignoni, M. Callieri, M. Corsini, M. Dellepiane, F. Ganovelli, G. Ranzuglia, Meshlab: An open-source mesh processing tool, in *Eurographics Italian Chapter Conference* (Salerno, Italy, 2008), vol. 2008, pp. 129–136.

71. G. Giribet, G. D. Edgecombe, The phylogeny and evolutionary history of arthropods. *Curr. Biol.* **29**, R592–R602 (2019).

72. B. Misof, S. Liu, K. Meusemann, R. S. Peters, A. Donath, C. Mayer, P. B. Frandsen, J. Ware, T. Flouri, R. G. Beutel, O. Niehuis, M. Petersen, F. Izquierdo-Carrasco, T. Wappler, J. Rust, A. J. Aberer, U. Aspöck, H. Aspöck, D. Bartel, A. Blanke, S. Berger, A. Böhm, T. R. Buckley, B. Calcott, J. Chen, F. Friedrich, M. Fukui, M. Fujita, C. Greve, P. Grobe, S. Gu, Y. Huang, L. S. Jermiin, A. Y. Kawahara, L. Krogmann, M. Kubiak, R. Lanfear, H. Letsch, Y. Li, Z. Li, J. Li, H. Lu, R. Machida, Y. Mashimo, P. Kapli, D. D. McKenna, G. Meng, Y. Nakagaki, J. L. Navarrete-Heredia, M. Ott, Y. Ou, G. Pass, L. Podsiadlowski, H. Pohl, B. M. von Reumont, K. Schütte, K. Sekiya, S. Shimizu, A. Slipinski, A. Stamatakis, W. Song, X. Su, N. U. Szucsich, M. Tan, X. Tan, M. Tang, J. Tang, G. Timelthaler, S. Tomizuka, M. Trautwein, X. Tong, T. Uchifune, M. G. Walzl, B. M. Wiegmann, J. Wilbrandt, B. Wipfler, T. K. F. Wong, Q. Wu, G. Wu, Y. Xie, S. Yang, Q. Yang, D. K. Yeates, K. Yoshizawa, Q. Zhang, R. Zhang, W. Zhang, Y. Zhang, J. Zhao, C. Zhou, L. Zhou, T. Ziesmann, S. Zou, Y. Li, X. Xu, Y. Zhang, H. Yang, J. Wang, J. Wang, K. M. Kjer, X. Zhou, Phylogenomics resolves the timing and pattern of insect evolution. *Science* **346**, 763–767 (2014).

73. R. Fernández, G. D. Edgecombe, G. Giribet, Phylogenomics illuminates the backbone of the Myriapoda Tree of Life and reconciles morphological and molecular phylogenies. *Sci. Rep.* **8**, 83 (2018).

74. G. D. Edgecombe, Chilopoda—Phylogeny, in *Treatise on Zoology - Anatomy, Taxonomy, Biology. The Myriapoda, Volume 1*, A. Minell, Ed. (Brill, 2011), pp. 339–354.

75. M. Rimsky-Korsakow, Ueber *Polyzonium germanicum* Brandt. *Proc. Imp. St-Petersburg Soc. Nat. Dep. Zool. Physiol.* **25**, 21–33 (1895).

76. T. Weichmann, T. Kleinteich, S. N. Gorb, B. Wipfler, Functional morphology of the mandibular apparatus in the cockroach *Periplaneta americana* (Blattodea: Blattidae) – a model species for omnivore insects. *Arthropod Systs. Phylogeny* **73**, 477–488 (2015).

77. J. Rodriguez, T. H. Jones, P. Sierwald, P. E. Marek, W. A. Shear, M. S. Brewer, K. M. Kocot, J. E. Bond, Step-wise evolution of complex chemical defenses in millipedes: A phylogenomic approach. *Sci. Rep.* **8**, 3209 (2018).

78. P. Sierwald, J. E. Bond, Current status of the myriapod class diplopoda (Millipedes): Taxonomic diversity and phylogeny. *Annu. Rev. Entomol.* **52**, 401–420 (2007).

79. H. Enghoff, S. I. Golovatch, A revision of the Siphonocryptidae (Diplopoda, Polyzoniida). *Zool. Scr.* **24**, 29–41 (1995).

80. R. M. Shelley, A revised, annotated, family-level classification of the Diplopoda. *Arthropoda Sel.* **11**, 187–207 (2003).

81. J. W. Shultz, Muscular anatomy of a whipspider, *Phrynus longipes* (Pocock) (Arachnida: Amblypygi), and its evolutionary significance. *Zool. J. Linn. Soc.* **126**, 81–116 (1999).

82. W. Meyer, Observations on the morphology and histochemistry of the foregut muscles of spiders (Arachnida: Araneida). *J. Morphol.* **170**, 113–131 (1981).

83. J. W. Shultz, Morphology of the prosomal endoskeleton of Scorpiones (Arachnida) and a new hypothesis for the evolution of cuticular cephalic endoskeletons in arthropods. *Arthropod Struct. Dev.* **36**, 77–102 (2007).

84. A. E. Klann, G. Alberti, Histological and ultrastructural characterization of the alimentary system of solifuges (Arachnida, Solifugae). *J. Morphol.* **271**, 225–243 (2010).

85. J. W. Shultz, Muscular anatomy of the giant whipscorpion *Mastigoproctus giganteus* (Lucas) (Arachnida: Uropygi) and its evolutionary significance. *Zool. J. Linn. Soc.* **108**, 335–365 (1993).

86. J. H. Swanepoel, A. Avenant-Oldewage, Functional morphology of the foregut of *Chonopeltis australis* Boxshall (Branchiura). *J. Crustac. Biol.* **13**, 656–666 (1993).

87. F. Karolyi, L. Morawetz, J. F. Colville, S. Handschuh, B. D. Metscher, H. W. Krenn, Time management and nectar flow: Flower handling and suction feeding in long-proboscid flies (Nemestrinidae: Prosoeca). *Naturwissenschaften* **100**, 1083–1093 (2013).

88. F. Karolyi, J. F. Colville, S. Handschuh, B. D. Metscher, H. W. Krenn, One proboscis, two tasks: Adaptations to blood-feeding and nectar-extracting in long-proboscid horse flies (Tabanidae, Philoliche). *Arthropod Struct. Dev.* **43**, 403–413 (2014).

89. K. Hamilton, Morphology and evolution of the rhynchotan head (Insecta: Hemiptera, Homoptera). *Can. Entomol.* **113**, 953–974 (1981).

90. J. A. Raven, Phytophages of xylem and phloem: A comparison of animal and plant sap-feeders, in *Advances in Ecological Research*, A. MacFadyen, E. D. Ford, Eds. (Academic Press, 1983), vol. 13, pp. 135–234.

91. S. C. Rastogi, The food pump and associated structures in *Coridius janus* (Fabr.) (Heteroptera: Dinidoridae). *Proc. R. Entomol. Soc. Lond. Ser. Gen. Entomol.* **40**, 125–134 (1965).

92. M. C. Parsons, Modifications of the food pumps of Hydrocorisae (Heteroptera). *Can. J. Zool.* **44**, 585–620 (1966).

93. M. R. Khan, The anatomy of the head-capsule and mouthparts of *Dysdercus fasciatus* Sign. (Pyrrhocoridae, Hemiptera). *J. Nat. Hist.* **6**, 289–310 (1972).

94. J. Davidson, On the mouth-parts and mechanism of suction in *Schizoneura lanigera, Hausmann*. *Zool. J. Linn. Soc.* **32**, 307–330 (1914).

95. H. Weber, Skelett, Muskulatur und Darm der schwarzen Blattlaus (*Aphis fabae* Scop.). *Fortschr. Zool.* **28**, 1–120 (1928).

96. M. Sorin, The mouth parts of *Aphis craccivora* Koch and the penetration of stylets into host plants. *Jap J Appl Ent Zool.* **5**, 217–224 (1961).

97. N. P. Kristensen, Phylogeny of endopterygote insects, the most successful lineage of living organisms. *Eur. J. Entomol.* **96**, 237–254 (1999).

98. L. E. S. Eastham, Y. E. E. Eassa, The feeding mechanism of the butterfly *Pieris brassicae* L. *Philos. Trans. R. Soc. Lond. B Biol. Sci.* **239**, 1–43 (1955).

99. F. Friedrich, H. Pohl, F. Beckmann, R. G. Beutel, The head of *Merope tuber* (Meropeidae) and the phylogeny of Mecoptera (Hexapoda). *Arthropod Struct. Dev.* **42**, 69–88 (2013).

100. M. I. Crichton, The structure and function of the mouth parts of adult caddis flies (Trichoptera). *Philos. Trans. R. Soc. Lond. B Biol. Sci.* **241**, 45–91 (1957).
